# 3D Lithiophilic Freestanding Hosts with SiO*_x_*‐Embedded Hierarchical Porous N‐Doped Carbon Nanofibers for Dendrite‐Free Lithium Metal Batteries

**DOI:** 10.1002/smll.202504223

**Published:** 2025-05-27

**Authors:** Yeon Woo Nahm, Jae Seob Lee, Jae Hun Choi, Jung Sang Cho, Yun Chan Kang

**Affiliations:** ^1^ Department of Materials Science and Engineering Korea University Anam‐Dong, Seongbuk‐Gu Seoul 136–713 Republic of Korea; ^2^ Department of Engineering Chemistry Chungbuk National University Cheongju Chungbuk 28644 Republic of Korea; ^3^ Biomedical Research Institute Chungbuk National University Hospital Cheongju Chungbuk 28644 Republic of Korea; ^4^ Advanced Energy Research Institute Chungbuk National University Cheongju Chungbuk 28644 Republic of Korea

**Keywords:** 3D host materials, electrospinning, freestanding, lithium metal battery, zeolitic imidazolate framework‐8

## Abstract

3D host materials are promising for Li‐metal anodes (LMAs) because of their adaptability to volume changes and large areas that prevent current localization, hindering dendritic Li formation. Herein, freestanding porous N‐doped carbon nanofibers (PCNFs) with uniformly distributed SiO*_x_* are synthesized by electrospinning and subsequent carbonization. In these composites, tunnel‐like, open channels are formed between the CNFs by removing polystyrene (PS) and the hollow N‐doped nanocages (HNC) generated from zeolitic imidazolate frameworks‐8 (ZIF‐8) during carbonization, providing sufficient space for Li deposition. Accompanying these structural advantages, the adequate electron conductivity and lithophilic properties derived from the conductive N‐doped CNFs and insulating SiO*_x_* confer optimized characteristics for uniform Li distribution. The coulombic efficiency exceeds 98% over 160 cycles in asymmetrical cells at a current density of 2.0 mA cm^−2^, with stable voltage hysteresis and an average overpotential of 25 mV for 1350 h in symmetrical tests. Full cells assembled with composite anodes predeposited with Li exhibited excellent capacity retention, delivering 141 mAh g^−1^ at 2.0 C with LiNi_0.6_Co_0.2_Mn_0.2_O_2_ (NCM622) cathodes. The results highlight that the optimized combination of conductive CNFs, HNCs, and insulating SiO*_x_* effectively enables uniform Li deposition, significantly enhancing cycling stability and Coulombic efficiency (CE) of LMAs.

## Graphical abstract

Herein, SiO*_x_* @PCNFs are synthesized by electrospinning process with subsequent thermal treatment. The SiO*_x_* @PCNFs showed tubular pores made by PS and HNC originating from ZIF‐8. The pore structures of these samples provided internal spaces for Li deposition while the optimized electrical conductivity is provided by conductive CNFs and insulative SiO*_x_* for uniform and dense Li distribution throughout the electrode. As a result of these advantages, SiO*_x_* @PCNFs showed enhanced electrochemical properties.

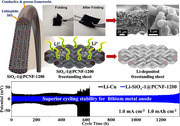

## 1 Introduction

With the rapid development of mobile electronics and electric vehicles, the need for high‐energy‐density battery systems has increased. Li‐metal anodes (LMAs) are promising owing to their high theoretical capacity (3860 mA h g^−1^), low potential (−3.04 V versus the standard hydrogen electrode), and low gravimetric density (0.534 g cm^−3^).^[^ ^1^, ^2^, ^3^, ^4^, ^5^ ^]^ However, the uncontrollable growth of Li dendrites and the generation of dead Li result in low Coulombic efficiency (CE), high internal resistance, short‐circuits, and explosion risks, restricting the practical application of LMAs.^[^ ^6^, ^7^ ^]^ Utilizing a 3D host has been reported to be an effective strategy because the host can provide sufficient space for Li deposition and suppress volume fluctuations.^[^ ^8^, ^9^, ^10^ ^]^ The high surface area of 3D hosts can also facilitate uniform Li ion flux and lower the local current density, which controls the growth of Li dendrites.^[^ ^11^, ^12^ ^]^

3D conductive frameworks such as Cu mesh, Ni foam, and carbon nanofibers (CNFs) are considered effective hosts for Li deposition.^[^ ^13^, ^14^, ^15^, ^16^ ^]^ CNFs are excellent, conductive host materials because of their highly interconnected fiber network, which shortens the path of motion of electrons and ions and promotes uniform Li distribution for more homogeneous Li plating.^[^ ^17^, ^18^, ^19^ ^]^ The gaps between the fibers not only alleviate the significant volume changes during long‐term electrochemical cycling but also provide enhanced flexibility, thereby improving the structural stability, preventing damage, and accommodating volume fluctuations in the electrodes.^[^ ^20^, ^21^ ^]^ This flexibility of CNFs enables their use as freestanding electrodes, allowing them to be directly employed without the need for a binder or current collector.^[^ ^22^, ^23^ ^]^ This simplifies the fabrication process, reduces costs, and lowers the interfacial resistance.^[^ ^24^ ^]^ Despite these advantages, the low affinity of CNFs for Li results in uneven Li nucleation and deposition at a given current density, leading to low CE and safety concerns.^[^ ^19^, ^25^ ^]^ N‐doped carbon enhances the lithiophilicity of carbon matrices and promotes uniform Li plating/stripping by reducing the Li nucleation overpotential through strong interactions with Lewis acidic Li ions in the electrolyte.^[^ ^21^, ^26^ ^]^ SiO_2_ can be electrochemically reduced to lithophilic Li*_x_* Si, which act as nanoseeds, lowering the nucleation potential and guiding the selective deposition of Li while inhibiting dendrite formation.^[^ ^27^, ^28^, ^29^ ^]^ The formation of CNF‐SiO_2_ composites prevents Li accumulation by redistributing the electric field, which enables uniform Li deposition. However, optimizing the electrode conductivity to achieve uniform Li plating by combining electroconductive and lithophilic insulating materials is an underexplored area of research, despite its significance. Furthermore, when the above strategy is combined with facile tuning of the pores of CNFs, novel structures that can facilitate uniform Li deposition can be synthesized.^[^ ^30^ ^]^

In this study, a 3D, freestanding, porous carbon nanofibers (PCNFs)‐SiO*_x_* composite with an optimized carbonization temperature and SiO*_x_* content, denoted as SiO*_x_*‐1@PCNF‐1200, is synthesized via electrospinning. The material is strategically designed as a freestanding electrode to eliminate the interfacial resistance between the current collector and the Li metal host structure, which is commonly present in conventional slurry‐type anodes. A 1D fibrous framework created via electrospinning provides the freestanding electrode with flexibility and mechanical integrity, ensuring structural stability during repeated lithium plating and stripping cycles. Within these fibers, we incorporated hierarchical, multiscale porous architecture to facilitate efficient Li ion transport and promote uniform Li accommodation throughout the host. Hollow N‐doped nanocages (HNCs), derived from size‐controlled zeolitic imidazolate framework‐8 (ZIF‐8) nanoparticles, and longitudinal channels formed by the decomposition of polystyrene (PS) enhance the lithiophilicity of the fibers while serving as internal reservoirs for lithium, enabling stable internal deposition. In addition, the optimized carbonization temperature and SiO*_x_* content ensure sufficient electrode conductivity, thereby facilitating uniform Li deposition across the entire electrode. Building on this stable Li deposition behavior, both asymmetrical and symmetrical cell tests demonstrate excellent cycle stability, where the performance is maintained even at high current densities. In addition, when paired with LiNi_0.6_Co_0.2_Mn_0.2_O_2_ (NCM622) and LiNi_0.8_Co_0.1_Mn_0.1_O_2_ (NCM811), the resulting full cells exhibit high cycle stability and superior rate performance, validating the practical viability of the SiO*_x_*‐1@PCNF‐1200 architecture.

## 2 Result and Discussion

**Scheme 1:**
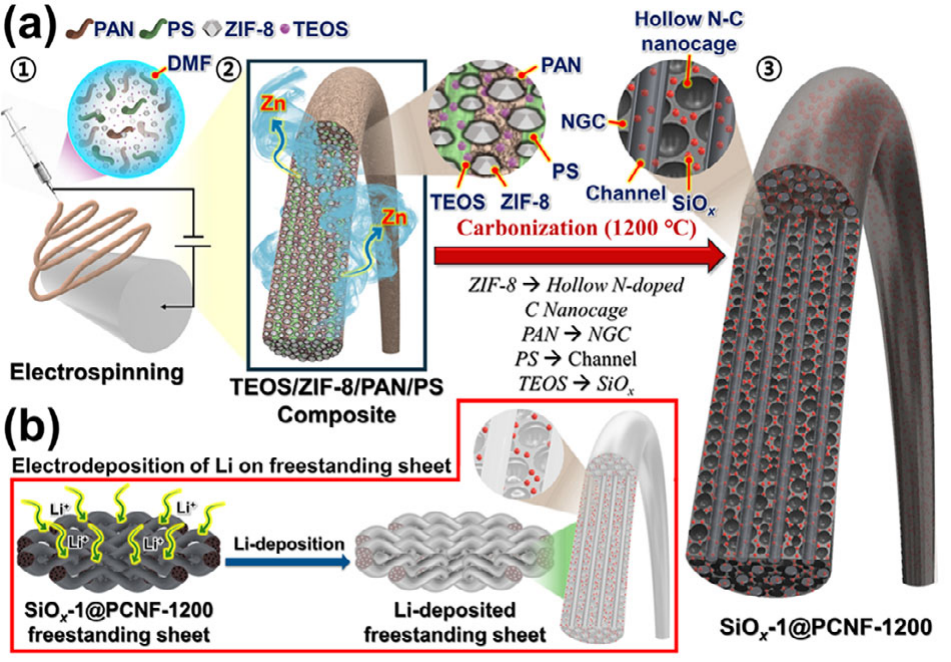
a) Formation mechanism of SiO*_x_*‐1@PCNF‐1200, b) Li deposition behaviors of SiO*_x_*‐1@PCNF‐1200.

**Scheme 2:**
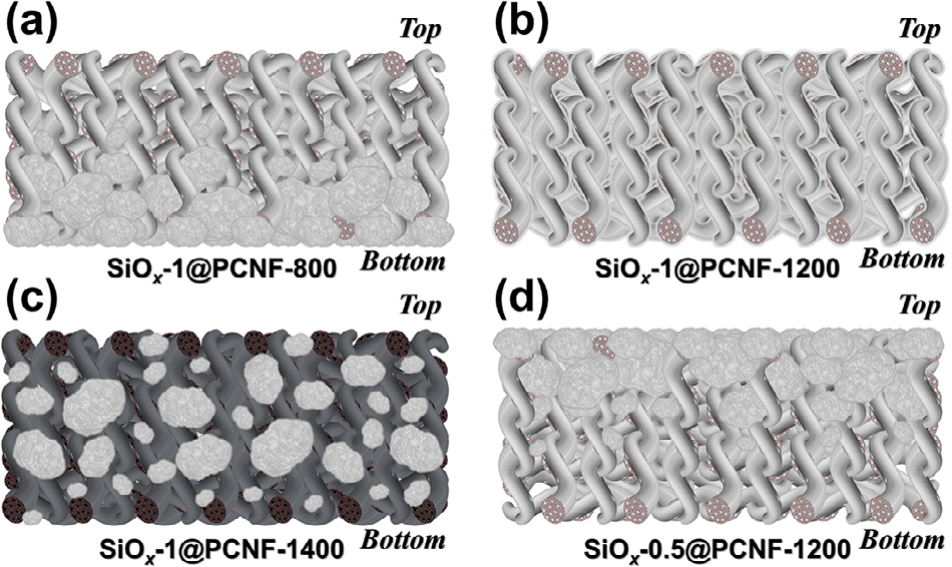
Schematic images of (a) SiO*_x_*‐1@PCNF‐800, b) SiO*_x_*‐1@PCNF‐1200, c) SiO*_x_*‐1@PCNF‐1400, and (d) SiO*_x_*‐0.5@PCNF‐1200 after Li deposition.

The detailed mechanism of formation of the SiO*_x_*‐1@PCNF‐1200 is illustrated in **Scheme** **1**. The prepared ZIF‐8 was mixed with tetraethyl orthosilicate (TEOS) as the SiO*_x_* source, polyacrylonitrile (PAN) as the N‐containing hard carbon source, and PS as the channel‐forming template in dimethylformamide (DMF) to form a well‐dispersed suspension, which was then electrospun (Scheme 1a‐①). Owing to the difference in their solubility parameters, PS (9.1 (cal cm^−3^)^1/2^) and PAN (12.5 (cal cm^−3^)^1/2^) did not form a compatible polymer blend, leading to phase separation in the spinning solution. As a result, PS formed a dispersed phase with an island‐like morphology, which aligned along the fiber length direction within the continuous TEOS/ZIF‐8/PAN composite phase under the influence of the electrical force during electrospinning. The resulting fibers were stabilized at 150 °C under an air atmosphere, yielding flexible, as‐spun fibers of the TEOS/ZIF‐8/PAN/PS composite (Scheme 1a‐②). The as‐spun fibers were then carbonized at 1200 °C under N_2_ atmosphere, where PAN decomposed and was transformed into N‐doped graphitic carbon and the dense ZIF‐8 particles were transformed into HNC with central mesopores. During the carbonization process, ZIF‐8 particles encapsulated within the carbon or polymer matrix underwent outward contractions, leading to the formation of HNC.^[^ ^31^, ^32^, ^33^ ^]^ Simultaneously, the Zn species within the ZIF‐8 framework were reduced to metallic Zn and evaporated during the subsequent high‐temperature annealing.^[^ ^34^, ^35^, ^36^ ^]^ Additionally, during the carbonization process, the thermal decomposition of TEOS facilitated the in situ formation of SiO_2_ within the nanofibers. Under high‐temperature conditions, SiO_2_ underwent partial carbothermal reduction, resulting in the formation of SiO*_x_* species. Furthermore, the dispersed PS phase stretched along the fiber length direction was completely thermally decomposed to gaseous materials, thereby forming numerous longitudinal channels within the CNFs, and ultimately leading to the formation of the final SiO*_x_*‐1@PCNF‐1200 (Scheme 1a‐③). Scheme 1b illustrates the electrochemical deposition of Li on a flexible freestanding sheet composed of SiO*_x_*‐1@PCNF‐1200. The HNCs, formed by the structural transformation of ZIF‐8, and longitudinal channels generated from PS decomposition, provide internal Li reservoirs and facilitate uniform Li distribution throughout the fiber. These structures, together with SiO*_x_*‐based lithiophilic sites and optimized electrode conductivity, contribute to dendrite suppression and stable Li deposition across the entire electrode.

**Figure 1:**
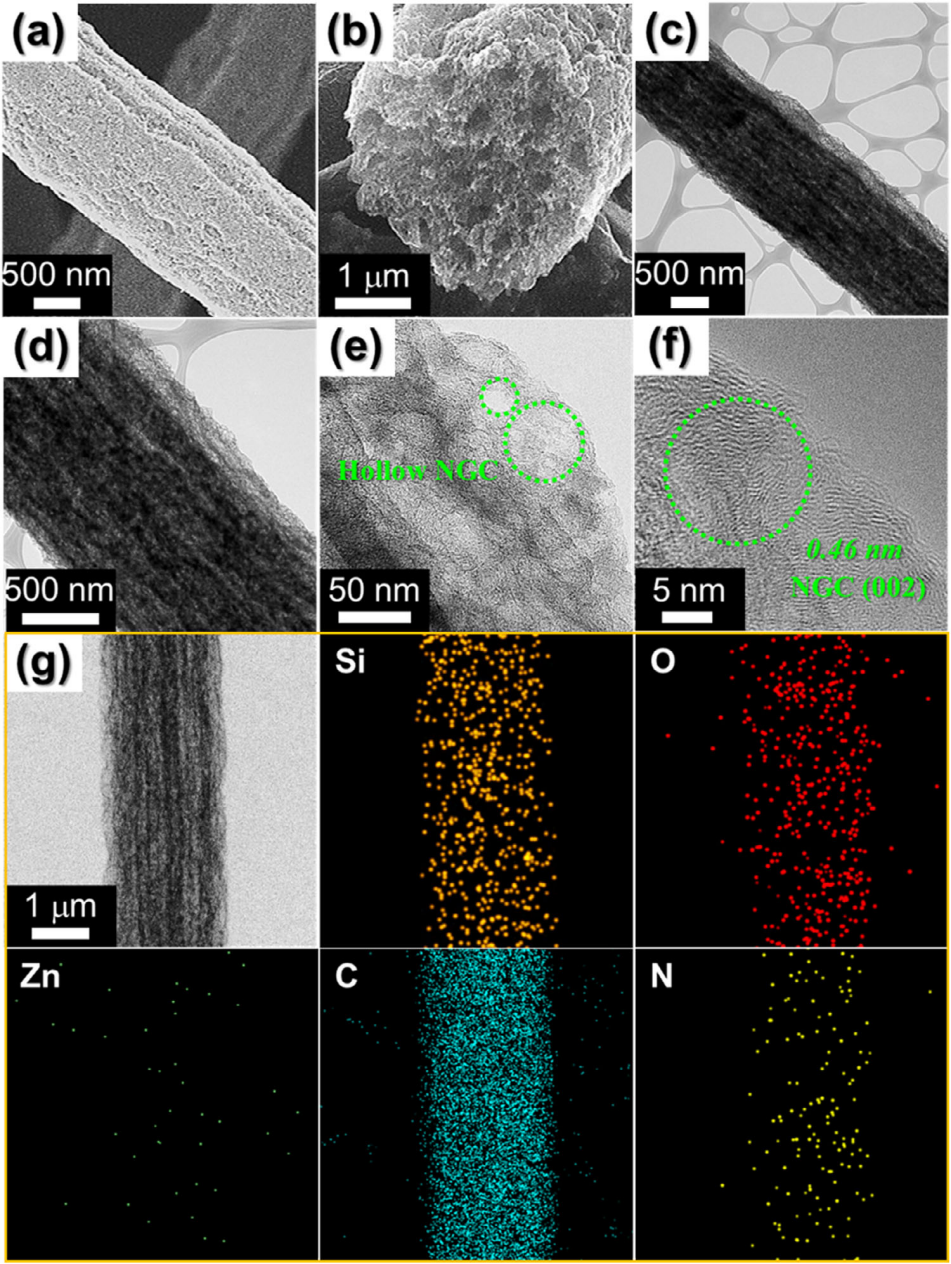
Morphologies and elemetal mapping images of the SiO*_x_*‐1@PCNF‐1200: a,b) FE‐SEM images, c–e) TEM images, f) HR‐TEM image, and (g) elemetmal mapping images.

To further understand the formation mechanism, the products obtained after each process were subjected to comprehensive morphological and crystal structure analyses. The morphologies of the ZIF‐8 polyhedra prepared for the synthesis of the composite fibers and the as‐spun TEOS/ZIF‐8/PAN/PS fibers were observed using field‐emission scanning electron microscope (FE‐SEM), as shown in Figure S1 (Supporting Information). The ZIF‐8 polyhedra prepared via liquid‐phase synthesis exhibited a uniform, polyhedral morphology with an average size of ≈40 nm (Figure S1a, Supporting Information). The as‐spun fibers synthesized by using these ZIF‐8 particles had a diameter of ≈2 µm, with rough surfaces containing embedded ZIF‐8 (Figure S1b,c, Supporting Information). The cross‐sectional view of the as‐spun fibers shown in Figure S1d (Supporting Information) reveals a dense fiber interior, where the stretched PS phase is observed as dispersed domains aligned along the fiber length direction, as indicated by the arrows. The X‐ray diffraction (XRD) spectra of the ZIF‐8 particles and as‐spun fibers exhibited the characteristic peaks of highly crystalline ZIF‐8, indicating that the ZIF‐8 structure remained intact during the spinning process (Figure S2, Supporting Information). The morphology and structural characteristics of the SiO*_x_*‐1@PCNF‐1200 obtained after carbonization of the as‐spun fibers are presented in **Figure** **1**. The FE‐SEM image of the carbonized fibers (Figure 1a) shows minimal changes on the fiber surface, whereas the image FE‐SEM in Figure 1b reveals that the previously dense cross‐section became significantly more porous. Transmission electron microscope (TEM) analysis revealed that the SiO*_x_*‐1@PCNF‐1200 exhibited an elongated, tubular, porous structure running along the fiber axis, forming a network of interconnected channels (Figure 1c). These tubular pores are formed by the phase‐separated PS polymers during electrospinning, which undergo thermal decomposition during carbonization, providing a distinctive anisotropic morphology within the fiber. The overall structural integrity of the remaining framework, composed of carbonized PAN, SiO*_x_* derived from TEOS, and the carbon structures derived from the ZIF‐8 particles, was maintained while allowing for continuous internal porosity. Closer examination of the magnified TEM image (Figure 1e) showed that the interconnected HNCs, ≈40 nm in size, contributed to the formation of the porous fiber structure. These HNCs were generated during the thermal process by carbonization of the organic ligands in ZIF‐8 and evaporation of metallic Zn, as well as by the string interaction between the PAN‐derived carbon species and ZIF‐8, which caused outward shrinkage, leaving a hollow, cage‐like structure. The high‐resolution transmission electron microscope (HR‐TEM) image in Figure 1f reveals that the lattice fringe of the carbon forming the HNCs is 0.46 nm, which is significantly larger than that (0.34 nm) typically observed for the (002) plane of graphitic carbon. The elemental mapping images (Figure 1g) confirm the uniform distribution of SiO*_x_* and C throughout the fiber, while also revealing the retention of N from PAN and ZIF‐8 despite the carbonization process. Zn was scarcely detected in the mapping images because of its low boiling point (907 °C), which led to its evaporation during high‐temperature heat treatment at 1200 °C.^[^ ^13^ ^]^ Thermogravimetry‐mass spectrometry (TG‐MS) was used to investigate the transformation of the as‐spun fibers into SiO*_x_*‐1@PCNF‐1200 during carbonization at 1200 °C under N_2_ atmosphere by analyzing the weight changes and evolved gases as a function of temperature (Figure S3, Supporting Information). The TG‐MS profile of the as‐spun fibers exhibited a sharp weight loss of 50% between ≈220 and 450 °C, primarily due to the decomposition of PAN, PS, and TEOS. During this stage, HCN gas was distinctly detected, originating from the breakdown of the nitrile (–C≡N) groups in PAN. Above 450 °C, continuous weight loss was observed, with a significant reduction in the HCN emissions, indicating the completion of PAN decomposition. The NO_2_ and CO_2_ emissions increased markedly above 550 °C, likely due to the further degradation of nitrogen‐ and carbon‐containing organic residues and ZIF‐8 decomposition. This suggests that above 450 °C, the carbonization of PAN‐derived organic compounds and ZIF‐8 led to the structural transformation into CNFs. As carbonization progressed, the NO_2_ and CO_2_ emissions decreased significantly beyond 950 °C, while gaseous Zn resulting from the evaporation of Zn became detectable.

To investigate the effect of the carbonization temperature on the fibers, the morphologies of the SiO*_x_*‐1@PCNF‐800 and ‐1400 synthesized at different carbonization temperatures using identical as‐spun fibers were compared (Figure S4, Supporting Information). Comparison of the FE‐SEM images of the SiO*_x_*‐1@PCNF‐800 and ‐1400 (Figure S4a,f, Supporting Information) shows that both samples exhibited similar surface and cross‐sectional morphologies, with no significant differences relative to that of SiO*_x_*‐1@PCNF‐1200 (Figure 1a,b). The TEM image of the SiO*_x_*‐1@PCNF‐1400 (Figure S4g, Supporting Information) revealed a pore structure nearly identical to that of the SiO*_x_*‐1@PCNF‐1200; however, the SiO*_x_*‐1@PCNF‐800 (Figure S4b, Supporting Information) did not exhibit the interconnected HNCs observed in the SiO*_x_*‐1@PCNF‐1200 and ‐1400, resulting in a darker central region in the TEM image of the former. The magnified TEM images (Figure S4c,h, Supporting Information) reveal that the SiO*_x_*‐1@PCNF‐1400 comprised distinct HNCs, whereas these structures were sparsely observed in the SiO*_x_*‐1@PCNF‐800. This discrepancy arises because the temperature of 800 °C is insufficient for complete carbonization, preventing the formation of well‐defined, HNCs. Owing to their relatively low carbonization temperature and lack of graphitic carbon, the HR‐TEM images of the SiO*_x_*‐1@PCNF‐800 exhibit a distinct lattice fringe of 0.25 nm, corresponding to the (002) plane of Zn (Figure S4d, Supporting Information). The HR‐TEM image of the SiO*_x_*‐1@PCNF‐1400 (Figure S4i, Supporting Information) shows a lattice fringe of 0.41 nm, associated with graphitic carbon, which, similar to that of the SiO*_x_*‐1@PCNF‐1200, is significantly larger than the lattice fringe of typical graphitic carbon. Elemental mapping images of the SiO*_x_*‐1@PCNF‐800 (Figure S4e, Supporting Information) also revealed a significant amount of Zn distributed within the fibers, unlike the SiO*_x_*‐1@PCNF‐1200 and ‐1400 (Figure 1g; Figure S4j, Supporting Information).

The FE‐SEM images of the SiO*_x_*‐0.5@PCNF‐1200 and SiO*_x_*‐1.5@PCNF‐1200, for which the SiO*_x_* content differed from that of the main sample (Figure S5a,f, Supporting Information) exhibit morphologies nearly identical to those of the main sample. However, the interior of the SiO*_x_*‐0.5@PCNF‐1200 (Figure S5b, Supporting Information) appeared relatively brighter than that of the main sample in the TEM images, whereas the interior of the SiO*_x_*‐1.5@PCNF‐1200 appeared darker (Figure S5g, Supporting Information). Comparison of the magnified TEM images revealed that the SiO*_x_*‐0.5@PCNF‐1200 exhibited clearly visible HNCs (Figure S5b, Supporting Information), whereas in the SiO*_x_*‐1.5@PCNF‐1200, the HNCs were obscured by the surrounding SiO*_x_* (Figure S5g, Supporting Information). Elemental mapping images of the distribution of Si also visually confirmed the difference in the SiO*_x_* content (Figure S5e and j, Supporting Information). The SiO*_x_* content, which was determined by the TEOS concentration, influenced the flexibility of the fibers. All the carbonized fibers, including SiO*_x_*‐1@PCNF‐1200, exhibited high flexibility, allowing them to recover their shape after folding and making them suitable as freestanding electrodes, as shown in Figure S6a–c (Supporting Information). However, the SiO*_x_*‐2.0@PCNF‐1200 with twice the TEOS concentration displayed significantly reduced flexibility, causing the electrode to fracture upon folding (Figure S6d–f, Supporting Information). This is attributed to the inherently higher stiffness and brittleness of SiO*_x_* compared to those of carbon fibers. As the SiO*_x_* content increased, the overall stiffness of the sheet increased, leading to greater resistance to tensile strain and, consequently, reduced flexibility.

**Figure 2:**
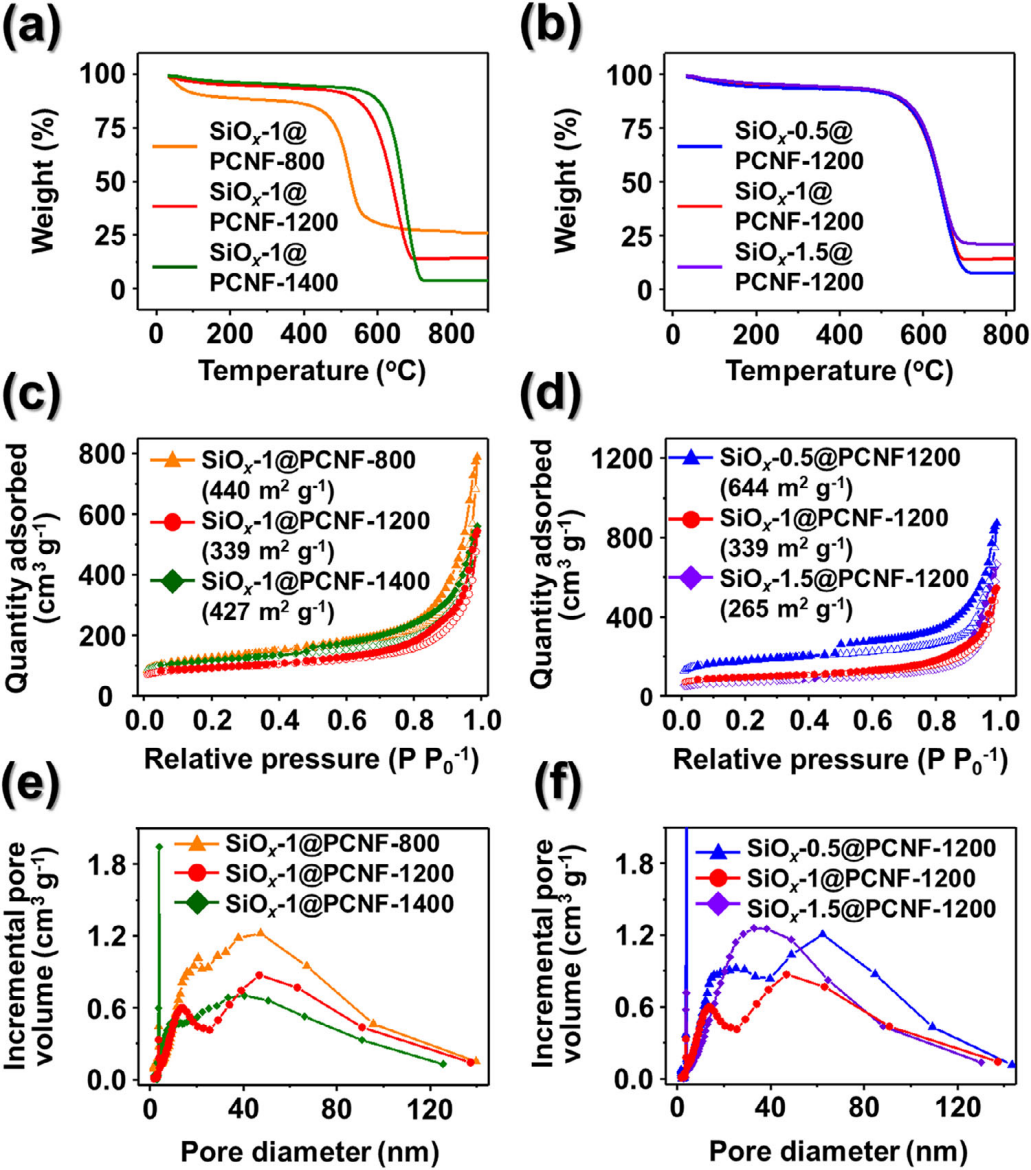
a,b) TGA curves, c,d) N_2_ adsorption and desorption isotherms, and (e,f) BJH desorption pore‐size distribution of various PCNFs. a,c,e) SiO*_x_*‐1@PCNFs‐800, ‐1200, and 1400, and (b,d,f) SiO*_x_*‐X@PCNFs‐1200 (X = 0.5, 1, 1.5).

XRD analysis was used to investigate the crystal structures of the synthesized fibers (Figure S7, Supporting Information). During carbonization of the as‐spun fibers containing ZIF‐8, graphitization was induced by the action of Zn as a catalyst, along with the graphitization of the PAN‐derived hard carbon. Consequently, the XRD spectra exhibited broad signals corresponding to the (002) and (101) planes of graphite, although not as sharp as those of highly crystalline graphite, suggesting partial graphitization. The presence of SiO*_x_* could not be clearly identified by XRD because of the amorphous nature of SiO*_x_*, which lacked well‐defined diffraction peaks. To determine the carbon and SiO*_x_* content in the synthesized fibers, thermogravimetric analysis (TGA) was conducted under air atmosphere; the results are presented in **Figure** **2**a,b. For the fibers synthesized at different carbonization temperatures (Figure 2a), the SiO*_x_*‐1@PCNF‐800 exhibited a sharp weight loss starting at 400 °C due to the combustion of carbon, whereas the SiO*_x_*‐1@PCNF‐1200 and ‐1400 underwent weight loss beginning at a relatively higher temperature of 500 °C. This shift to a higher combustion temperature indicates that the interaction energy between the carbon atoms increases with increasing carbonization temperature. Thus, the PCNFs synthesized at higher temperatures contain a greater proportion of graphitic carbon compared to those synthesized at 800 °C, which suggests improved structural ordering and higher thermal stability of the former. After the weight loss induced by carbon combustion, the weight stabilized above 700 °C, corresponding to the amount of residual material, which was calculated as 26.2, 14.6, and 3.9 wt.% for the SiO*_x_*‐1@PCNF‐800, ‐1200, and ‐1400, respectively. The higher amount of residue from the SiO*_x_*‐1@PCNF‐800 compared to the SiO*_x_*‐1@PCNF‐1200 does not indicate a genuinely higher SiO*_x_* content but rather results from the residual Zn in the former due to the lower carbonization temperature, which was subsequently converted to ZnO during the TGA, contributing to the additional weight of the SiO*_x_*‐1@PCNF‐800. On the other hand, the particularly low amount of residue from the SiO*_x_*‐1@PCNF‐1400 is attributed to SiO*_x_* sublimation at the high carbonization temperature of 1400 °C.^[^ ^37^ ^]^ The TGA data for the fibers with varying SiO*_x_* contents (Figure 2b) show that the weight loss induced by carbon combustion began at the same temperature for all samples owing to identical carbonization conditions. For the fibers with varying SiO*_x_* contents, the SiO*_x_* content was calculated using the same method, yielding values of 21.7, 14.6, and 8.0 wt.%, which correspond proportionally to the TEOS content.

The pore structure and surface area of the synthesized fibers were analyzed using Brunauer‐Emmett‐Teller (BET) measurements (Figure 2c–f). The nitrogen gas adsorption–desorption isotherms of all samples were classified as type IV, indicating the presence of mesopores (Figure 2c,d). The surface area calculated using the BET method was 440, 339, 427, 644, and 265 m^2^ g^−1^ for the SiO*_x_*‐1@PCNF‐800, SiO*_x_*‐1@PCNF‐1200, SiO*_x_*‐1@PCNF‐1400, SiO*_x_*‐0.5@PCNF‐1200, and SiO*_x_*‐1.5@PCNF‐1200, respectively. With increasing carbonization temperature, the surface area decreased from 800 to 1200 °C but increased again at 1400 °C. This trend is attributed to the transformation of the microporous ZIF‐8 into HNC during carbonization, which led to a reduction in the number of micropores. At 800 °C, this transformation was incomplete, resulting in a larger surface area than that of the SiO*_x_*‐1@PCNF‐1200. In contrast, the SiO*_x_*‐1@PCNF‐1400 exhibited an increased surface area owing to the sublimation of SiO*_x_* at high temperatures, which reduced the proportion of SiO*_x_*, a denser component with a lower surface area than carbon, ultimately contributing to an overall increase in the surface area. The decrease in the surface area with increasing SiO*_x_* content (Figure 2d) can be explained by the same reasoning. The mesopore size distribution analyzed using the Barrett–Joyner–Halenda (BJH) method (Figure 2e,f) indicated that all samples contained pores of various sizes within the mesopore range, which contributed to their high surface areas.

X‐ray photoelectron spectroscopy (XPS) was employed to investigate the chemical composition and bonding states of the different elements in SiO*_x_*‐1@PCNF‐1200. The survey spectrum in Figure S8a (Supporting information) shows photoelectron signals corresponding to Si 2*p*, O 1*s*, C 1*s*, and N 1*s*, indicating the successful incorporation of SiO*_x_* within the N‐doped carbon nanofiber matrix. The high‐resolution Si 2*p* core‐level XPS profile (Figure S8b) displays peaks at 103.0 and 103.5 eV, which correspond to the Si–O/Si–O–C and SiO_2_ bonding states, confirming the presence of SiO*_x_* species within the composite nanofibers.^[^ ^38^ ^]^ Notably, the intensity of the Si–O/Si–O–C peak was higher than that of the SiO_2_ peak, attributed to the partial carbothermal reduction of SiO_2_ at high carbonization temperatures. This reduction process results in the formation of Si–O species because of the strong interactions between SiO*_x_* and the surrounding carbon fibers. Additionally, the fitted peak at 101.1 eV is assigned to Si–C bonds, further highlighting the robust interfacial interaction between SiO*_x_* and the surrounding carbonaceous material.^[^ ^38^, ^39^, ^40^ ^]^ The O 1*s* XPS profiles (Figure S8c, Supporting Information) further confirm the presence of SiO*_x_* and oxygen‐functionalized carbon species, which may enhance the surface reactivity.^[^ ^39^, ^41^ ^]^ The deconvoluted C 1*s* spectrum (Figure S8d, Supporting information) exhibits six peaks corresponding to various carbon‐bonding configurations, including C–Si (283.9 eV), C–C (sp^2^) (284.5 eV), C–C (sp^3^)/C–N (285.4 eV), C–O (287.0 eV), C═O (288.7 eV), and O–C═O (290.6 eV).^[^ ^38^, ^42^ ^]^ The dominance of the C–C (sp^2^) peak underscores the formation of highly conductive graphitic carbon, which originates from graphitization catalyzed by Zn, as well as the PAN‐derived hard‐carbon component.^[^ ^43^ ^]^ Furthermore, the presence of the C–Si bond provides additional evidence of the strong interfacial interactions between carbon and SiO*_x_*, reinforcing the structural stability of the composite fibers. The C–C (sp^3^)/C–N peak confirmed successful N‐doping within the carbon framework, further enhancing the electronic properties.^[^ ^42^ ^]^ The N 1*s* XPS profile (Figure S8e, Supporting Information) provided additional evidence of N‐doping, revealing four distinct peaks assigned to pyridinic N (398.1 eV), pyrrolic N (399.7 eV), graphitic N (401.0 eV), and oxidized N (403.0 eV).^[^ ^42^, ^44^ ^]^ The well‐defined high‐resolution C 1*s* and N 1*s* spectra strongly support the formation of a N‐doped graphitic carbon matrix within the nanofiber structure. Raman spectroscopy was used to analyze the carbon characteristics of the structure of SiO*_x_*‐1@PCNFs carbonized at different temperatures (800, 1200, and 1400 °C) (Figure S8f, Supporting Information). The three samples exhibited characteristic D‐ and G‐bands at 1344 and 1589 cm^−1^, indicating the presence of disordered and graphitic carbon structures, respectively.^[^ ^45^, ^46^ ^]^ The peak at 2685 cm^−1^ corresponds to the 2D‐band, which is attributed to the overtone of the D‐band and results from the double‐resonance effect related to the bonding structure of graphitic carbon.^[^ ^47^ ^]^ The intensity ratio of the D‐ to G‐band (*I*_D_/*I*_G_), a key parameter for assessing the crystallinity of carbon, varied among the samples, where the *I*_D_/*I*_G_ ratio for SiO*_x_*‐1@PCNF‐800 was 1.13, indicating the predominantly amorphous nature of carbon. However, with increasing carbonization temperature, the *I*_D_/*I*_G_ ratio gradually decreased to 1.06 and 1.04 for SiO*_x_*‐1@PCNF‐1200 and ‐1400, respectively, signifying enhanced graphitization. This is attributed to the catalytic effect of Zn on the graphitization process, as well as the properties of the PAN‐derived hard carbon, which facilitate the transition toward a more ordered carbon structure. Based on the XPS and Raman results, these findings collectively demonstrate that lithiophilic SiO*_x_* was homogeneously embedded within the N‐doped CNF network, whereas improved graphitization at elevated temperatures enhanced the electrical conductivity and structural stability, both of which are expected to improve the Li deposition properties and overall performance of SiO*_x_*‐1@PCNF‐1200 as a Li host.

**Figure 3:**
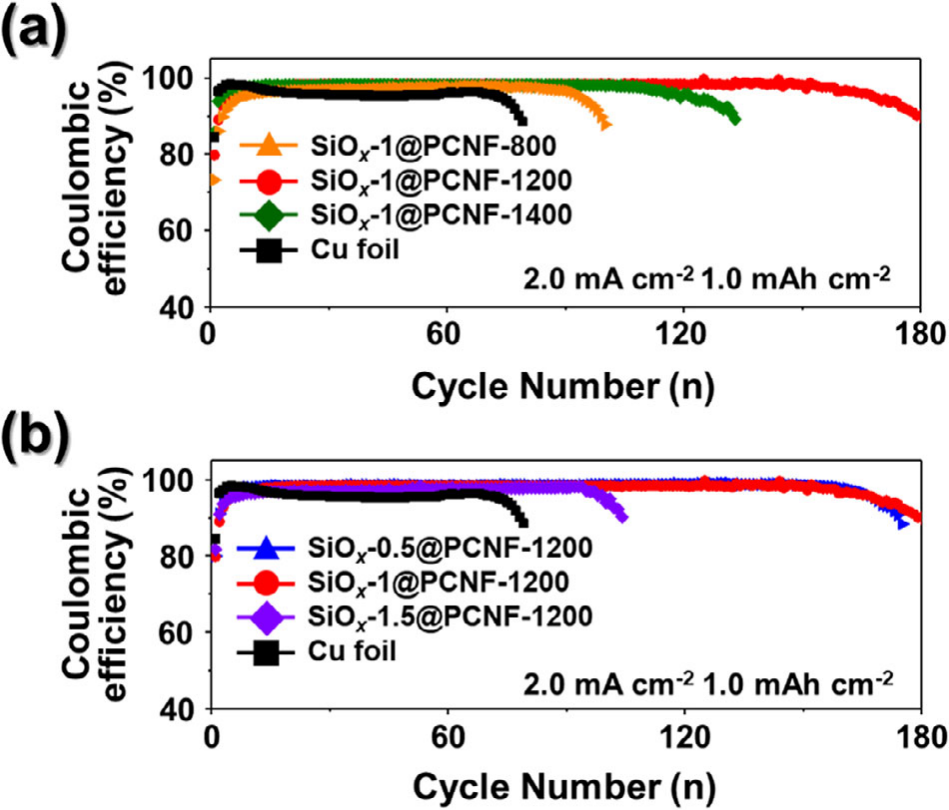
CEs of (a) SiO*_x_*‐1@PCNFs‐800, ‐1200, ‐1400, and bare Cu foil, and (b) SiO*_x_*‐X@PCNFs‐1200 (X = 0.5, 1, 1.5) and bare Cu foil at a current density of 2.0 mA cm^−2^ with a cycling capacity of 1.0 mAh cm^−2^.

To evaluate the electrochemical properties of the SiO*_x_* @PCNFs, asymmetric cell tests were conducted using the SiO*_x_* @PCNFs as the working electrode and Li foil as the counter electrode. These tests were used to compare the stability and CE of the SiO*_x_* @PCNFs during repeated Li deposition and stripping cycles. The CE was calculated as the ratio of the capacity of the deposited Li to the capacity of the Li stripped during the process, where this value strongly reflects the stability of the Li‐metal anode. First, the effects of the carbonization temperature were examined by conducting asymmetric cell tests using SiO*_x_* @PCNFs synthesized with a fixed TEOS concentration while varying the carbonization temperature (**Figure** **3**a). The asymmetrical cell tests were conducted at a current density of 2.0 mA cm^−2^ with a total areal capacity of 1.0 mAh cm^−2^. Among the samples carbonized at different temperatures, SiO*_x_*‐1@PCNF carbonized at 1200 °C (SiO*_x_*‐1@PCNF‐1200) demonstrated the highest cycling stability, maintaining a CE of 98% over 160 cycles. SiO*_x_*‐1@PCNFs‐800 and ‐1400 exhibited CEs of 97% and 98% over 85 and 100 cycles, respectively, outperforming Cu foil, which afforded a CE of 95% after 70 cycles. However, both fibers demonstrated lower electrochemical performance than the SiO*_x_*‐1@PCNF‐1200. To compare the characteristics based on the SiO*_x_* content, the carbonization temperature was fixed at 1200 °C, and the effect of the TEOS concentration on the properties was analyzed (Figure 3b). Among the samples synthesized with various TEOS concentrations, the baseline SiO*_x_*‐1@PCNF‐1200 and SiO*_x_*‐0.5@PCNF‐1200 synthesized with half the TEOS concentration both exhibited nearly identical performance, maintaining a CE exceeding 98% over 160 cycles. However, doubling the TEOS concentration in the SiO*_x_*‐1.5@PCNF‐1200 resulted in a lower CE of 97.5% during the initial 60 cycles, followed by an increase to 98% at the 92th cycle, after which the cells failed. The SiO*_x_*‐X@PCNFs‐1200 (X = 0.5, 1) fibers carbonized at 1200 °C demonstrated the most stable performance during repeated cycling, along with reversible Li deposition and stripping characteristics. To further highlight the superior cyclability and highly reversible Li plating/stripping behavior of the freestanding SiO*_x_*‐1‐PCNFs‐1200 electrode, a comparison of asymmetric cell data of recent studies utilizing carbon nanofibers or SiO_2_‐based structures as Li metal hosts is summarized in Table S1 (Supporting Information). These findings highlight the superior electrochemical stability of the SiO*_x_*‐1@PCNFs‐1200 fibers, although further analysis is required to comprehensively evaluate their electrochemical properties.

**Figure 4:**
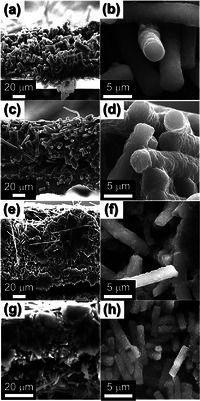
Cross‐sectional SEM images of (a,b) SiO*_x_*‐1@PCNF‐800, c,d) SiO*_x_*‐1@PCNF‐1200, e,f) SiO*_x_*‐1@PCNF‐1400, and (g,h) SiO*_x_*‐0.5@PCNF‐1200 after Li deposition with total capacity of 5.0 mAh cm^−2^ at 2.0 mA cm^−2^ for each electrode.

For an in‐depth understanding of the performance of the fibers in an asymmetric cell, the Li deposition behavior of the SiO*_x_* @PCNF was examined using SEM after plating 5.0 mAh cm^−2^ of Li at a current density of 2.0 mA cm^−2^ (**Figure** **4**). The cross‐sectional SEM images of SiO*_x_*‐1@PCNF‐800 and ‐1200 after Li deposition, shown in Figure 4a,b respectively, reveal that Li was deposited along the surface of the fibers in both samples. Compared to the porous cross‐section of the fibers before deposition (Figure 1b), the post‐deposition cross‐section appeared densely filled, indicating that Li was deposited not only on the fiber surface but also within the pores of the fibers (Figure 4e,f). The SiO*_x_*‐1@PCNF‐800 and ‐1200 had initial diameters of 1.8 and 1.6 µm before Li deposition, which increased to 5.2 and 4.7 µm, respectively, after deposition (Figure 4e,f). This observation suggests that the SiO*_x_*‐1@PCNF‐800, ‐1200 exhibited a capacity for storing Li within the fiber structure. Although Li was deposited within the pores and on the fiber surface of both samples, Li accumulated locally in the SiO*_x_*‐1@PCNF‐800, predominantly in the lower regions of the electrode, as indicated by the dotted lines in Figure 4a. In contrast to the SiO*_x_*‐1@PCNF‐800 and ‐1200, the thickness of the SiO*_x_*‐1@PCNF‐1400 exhibited no noticeable changes before and after Li deposition. Instead, Li predominantly accumulated in a relatively bulky form within the inter‐fiber spaces (Figure 4e,f). This deposition behavior led to significantly greater volume expansion in the SiO*_x_*‐1@PCNF‐1400 electrode than in the SiO*_x_*‐1@PCNF‐800 and ‐1200, which can be attributed to the localized accumulation of Li primarily between the fibers rather than being stored within their structures. Bulk Li deposition was observed in both the SiO*_x_*‐0.5@PCNF‐1200 and SiO*_x_*‐1@PCNF‐1400; however, the deposition behaviors were distinctly different (Figure 4f,h). In the SiO*_x_*‐0.5@PCNF‐1200, Li was initially deposited on the fiber surfaces, followed by the formation of bulk Li that completely covered the fibers. In contrast, the SiO*_x_*‐1@PCNF‐1400 predominantly exhibited bulk Li deposition confined to the spaces between the fibers, with minimal surface coverage. Another distinction from the SiO*_x_*‐1@PCNF‐1400 is that the bulk Li was localized on the upper portion of the electrode rather than being distributed across the entire electrode. The lower section of the SiO*_x_*‐0.5@PCNF‐1200 electrode exhibited comparatively dendritic Li growth (Figure 4h), unlike the more uniform deposition observed for the SiO*_x_*‐1@PCNF‐800 and ‐1200 electrodes. To clarify the distinct Li deposition behavior resulting from the characteristics of the synthesized fibers, illustrations are presented in **Scheme** **2**.

The difference in the transfer rate of the Li ions versus the electrons causes the scaffolds with higher electrical conductivities to preferentially facilitate Li deposition in the upper regions.^[^ ^48^ ^]^ To explain the difference in the Li deposition process with the various SiO*_x_* @PCNFs, the electrical conductivities were compared using a four‐point probe, and the results are summarized in Table S2 (Supporting Information). Comparison of the electrical conductivity of the fibers carbonized at different temperatures (SiO*_x_*‐1@PCNF‐800, ‐1200, and ‐1400) showed that the resistance of the SiO*_x_*‐1@PCNF‐800 fibers was too high to be measured. The resistance of the SiO*_x_*‐1@PCNF‐1200 and ‐1400 was 0.73 and 2.32 S cm^−1^, respectively. These results are consistent with those of the Raman analysis shown in Figure S8f (Supporting Information), where the high resistance of SiO*_x_*‐1@PCNF‐800 is attributed to its lower proportion of conductive graphitic carbon compared to that of the SiO*_x_*‐1@PCNF‐1200 and ‐1400. The higher conductivity of the SiO*_x_*‐1@PCNF‐1400, despite having an *I_D_/I_G_* ratio similar to that of the SiO*_x_‐*1@PCNF‐1200 can be explained by the sublimation of SiO*_x_* at the elevated temperature of 1400 °C, which reduces the content of insulative SiO*_x_*. Based on the SiO*_x_* content, the SiO*_x‐_* X@PCNFs‐1200 (X = 0.5, 1, 1.5) exhibited electrical conductivities of 1.9, 0.73, and 0.42 S cm^−1^, respectively, where the conductivity decreased with increasing SiO*_x_* content. This analysis of the electrical conductivity explains the localized Li deposition in the lower regions of the low‐conductivity SiO*_x_*‐1@PCNF‐800, and the deposition of Li in the upper region in the high‐conductivity SiO*_x_*‐0.5@PCNF‐1200. By blending the carbonized fibers having high electrical conductivity with insulating SiO*_x_*, the conductivity can be modulated to enable uniform Li deposition on the electrode. However, the role of SiO*_x_* in SiO*_x_* @PCNF is limited solely to reducing the electrical conductivity to prevent Li accumulation.

To address the above question, cyclic voltammetry (CV) data were acquired for the SiO*_x_*‐1@PCNF‐800, SiO*_x‐_* 1@PCNF‐1200, and SiO*_x_*‐0.5@PCNF‐1200 at scan rates ranging from 0.1 to 2.0 mV s^−1^. All samples (Figure S9a–c, Supporting Information) exhibited distorted rectangular shapes with broad redox peaks, indicative of combined capacitive and faradaic charge storage.^[^ ^49^ ^]^ The cathodic and anodic peaks, observed near 0.0 and 0.2 V, respectively, reflect reversible redox reactions likely involving Li interactions with polar SiO*_x_* and N‐doped carbon domains.^[^ ^50^ ^]^ To further elucidate the charge storage mechanism, the relationship between the peak current (*i*) and scan rate (*v*) was analyzed using the equation *i = a·v^b^*. A b‐value near 0.5 denotes a diffusion‐controlled process, whereas a value near 1.0 implies surface‐controlled capacitive behavior. The calculated b‐values for the cathodic scans of SiO*_x_*‐1@PCNF‐800, SiO*_x_*‐1@PCNF‐1200, and SiO*_x_*‐0.5@PCNF‐1200 were 0.76, 0.65, and 0.72, respectively, while the anodic scans yielded values of 0.84, 0.83, and 0.90, respectively (Figure S9d,e, Supporting Information). These results confirm that both capacitive and diffusion‐controlled processes contribute to the overall charge storage. This quantitative analysis aligns well with the CV shape features, supporting a hybrid charge storage mechanism in the composite electrodes.^[^ ^51^ ^]^ Additionally, the cyclic voltammograms were used to calculate the areal capacitance of each material. At a scan rate of 0.1 mV s^−1^, the capacitance of the SiO*_x_*‐1@PCNF‐800, SiO*_x_*‐1@PCNF‐1200, and SiO*_x_*‐0.5@PCNF‐1200 was 1.07, 1.51, and 1.15 F cm^−2^, respectively (Figure S9f, Supporting Information). At all scan rates, the SiO*_x_*‐1@PCNF‐1200 exhibited the highest capacitance, indicating a higher concentration of Li ions near the surface of the structure. According to Sand's time theory, dendritic Li growth occurs when the concentration of Li ions approaches zero; therefore, the high capacitance at elevated current densities suggests that the SiO*_x_*‐1@PCNF‐1200 can prevent dendritic Li growth and facilitate uniform Li deposition.^[^ ^52^ ^]^ Although the capacitance of the composite structures was generally proportional to the surface area and electrical conductivity, the SiO*_x_*‐1@PCNF‐1200 exhibited the highest capacitance despite having the lowest surface area measured by BET and a lower electrical conductivity than the SiO*_x_*‐0.5@PCNF‐1200 with less SiO*_x_*. This phenomenon can be attributed to the high polarity of SiO*_x_* the negatively charged oxygen atoms on the surface of SiO*_x_* enable strong electrostatic interactions with Li ions, resulting in stable chemical adsorption that outweighs physical adsorption.^[^ ^53^ ^]^ In contrast, nonpolar carbon lacks the capacity for chemical bonding and primarily interacts via weaker physical adsorption, which limits its ability to stably anchor Li ions. This balance of conductive graphitic carbon and polar SiO*_x_* in the SiO*_x_*‐1@PCNF‐1200 allowed for uniform and dense Li deposition, even at a high capacity of 5.0 mAh cm^−2^, where Li was primarily deposited within the internal pores and surrounding surfaces of the fibers. The deposition of bulk Li observed in the SEM images of the SiO*_x_*‐0.5@PCNF‐1200 and SiO*_x_*‐1@PCNF‐1400 (Figure 4g,e), which have lower SiO*_x_* contents, can also be explained by the polarity of SiO*_x_* and carbon.

To further elucidate the role of SiO*_x_* in SiO*_x_*‐1@PCNF‐1200, a comparative in situ electrochemical impedance spectroscopy (EIS) analysis was performed on SiO*_x_*‐1@PCNF‐1200 and the sample without SiO*_x_* denoted as SiO*_x_*‐0@PCNF‐1200, and as presented in Figure S10 (Supporting Information). The in situ EIS measurements were proceeded during Li plating and stripping process at a current density of 1.0 mA cm^−2^, corresponding to a capacity of 1.0 mAh cm^−2^ for each step. Each plating and stripping cycle lasted 1 h and was segmented into six intervals, with EIS data collected every 10 min. The voltage profile in Figure S10a (Supporting Information) illustrates the timing of EIS measurements throughout the plating and stripping process. The colored dots in Figure S10a (Supporting Information) represent the specific time points at which EIS measurements were performed, while the corresponding EIS spectra are plotted in Figure S10b–e (Supporting Information) using the same color scheme to indicate each measurement step. The resistance data from the EIS analysis was separated into three components: The electrolyte resistance (*R*_ele_) is defined by the starting point of the semicircle, the solid electrolyte interphase (SEI) resistance (*R*_SEI_) corresponds to the semicircle in the high‐frequency region, and the charge‐transfer resistance (*R*_ct_) is represented by the semicircle in the middle‐frequency region.^[^ ^54^ ^]^ Both SiO*_x_*‐0@PCNF‐1200 and SiO*_x_*‐1@PCNF‐1200 electrodes exhibit similar initial *R*_ele_ values. During the Li plating process *R*_ct_ values increase (Figure S10b,d, Supporting Information) and then subsequently decrease during the stripping process (Figure S10c,e, Supporting Information). This behavior is consistent with typical Li metal cell characteristics, where the resistance increases during Li deposition owing to SEI formation and morphological changes and subsequently decreases during removal of Li.^[^ ^55^, ^56^, ^57^ ^]^ However, compared to SiO*_x_*‐0@PCNF‐1200, SiO*_x_*‐1@PCNF‐1200 exhibited a noticeably slower resistance increase during Li plating. This improvement is attributed to the uniformly distributed lithiophilic SiO*_x_* within the electrode, which promotes uniform and dense Li deposition, thereby mitigating the typical resistance escalation during cycling.

**Figure 5:**
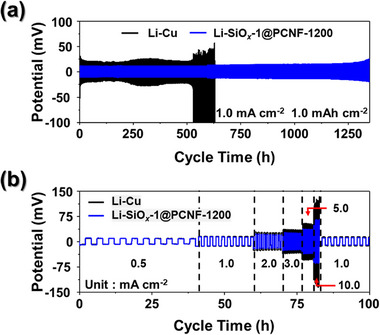
Results of symmetrical cell test of Li‐Cu and SiO*_x_*‐1@PCNF‐1200 electrodes; a) cycling performance at 1.0 mA cm^−2^ with a capacity of 1.0 mA h cm^−2^, and (b) rate performance measured at current density from 0.5 to 10.0 mA cm^−2^ with a fixed capacity of 1.0 mA h cm^−2^.

Symmetric cell tests were conducted to evaluate the practical performance of the SiO*_x_*‐1@PCNF‐1200 as Li host structures for LMAs. The tests were performed by pre‐depositing 5.0 mAh cm^−2^ of Li onto Cu foil and the SiO*_x_*‐1@PCNF‐1200, designated as Li‐Cu and Li‐SiO*_x_*‐1@PCNF‐1200, respectively. The results are presented in **Figure** **5**. To compare the long‐term stability of the Li‐Cu and Li‐SiO*_x_*‐1@PCNF‐1200 symmetric cells, cycle tests were conducted at a current density of 1 mA cm^−2^ with a capacity of 1.0 mAh cm^−2^ (Figure 5a). The Li‐Cu symmetric cell initially exhibited a voltage hysteresis of 340 mV, which remained stable for 200 h before increasing to 2820 mV and failing before 550 h. In contrast, the Li‐SiO*_x_*‐1@PCNF‐1200 symmetric cell utilizing the freestanding electrodes demonstrated an initial small voltage hysteresis of 23 mV, which was maintained for over 600 h. Subsequently, voltage hysteresis increased slightly to 28 mV and remained stable for over 1350 h, before a gradual increase in resistance was observed. The rate capabilities of symmetric cells were tested at various current densities ranging from 0.5 to 10 mA cm^−2^, maintaining a fixed capacity of 1 mAh cm^−2^ (Figure 5b). The Li‐Cu symmetric cell exhibited voltage hysteresis values of 16, 29, 51, 66, 102, and 203 mV when the current density was increased to 0.5, 1.0, 2.0, 3.0, 5.0, and 10.0 mA cm^−2^, respectively, whereas the voltage hysteresis of the Li‐SiO*_x_*‐1@PCNF‐1200 symmetric cell was quantified as 15, 24, 37, 45, 69, and 123 mV under the same conditions. At current densities of 1.0 mA cm^−2^ or lower, the resistance of both cells was nearly identical. However, as the current density increased, the resistance of the Li‐Cu symmetric cell increased significantly, resulting in a voltage hysteresis difference exceeding 100 mV at the high current density of 10 mA cm^−2^. The results of the symmetric cell tests demonstrated that the SiO*_x_*‐1@PCNF‐1200, which enables uniform and dense lithium deposition as a 3D host structure, could serve as stable and low‐resistance LMAs, even after loading a significant amount of Li. In order to underscore the outstanding symmetric cell performance, including low voltage hysteresis and long‐term cycling stability of the SiO*_x_*‐1@PCNF‐1200 electrode, a comparison with previously reported Li metal hosts employing similar structures was conducted, as shown in Table S3 (Supporting Information). Notably, while other comparative materials exhibited significant increases in hysteresis voltage at higher current densities, the SiO*_x_*‐1@PCNF‐1200 electrode maintained a remarkably stable voltage profile, reflecting its enhanced Li hosting capability.

EIS was used to investigate whether the use of the SiO*_x_*‐1@PCNF‐1200 as a freestanding electrode contributed to lowering the electrochemical resistance. The resistance of the SiO*_x_*‐1@PCNF‐1200 freestanding electrode was compared with that of the SiO*_x_*‐1@PCNF‐1200 cast onto Cu foil, denoted as Cu‐SiO*_x_*‐1@PCNF‐1200. The resistance of the freestanding SiO*_x_*‐1@PCNF‐1200 and Cu‐SiO*_x_*‐1@PCNF‐1200 electrodes before and after lithiation was compared (Figure S11, Supporting Information). Before lithiation, the freestanding SiO*_x_*‐1@PCNF‐1200 and Cu‐SiO*_x_*‐1@PCNF‐1200 electrodes exhibited identical *R*_ele_ values. However, the total resistance (*R*_tot_), defined as the sum of *R*_SEI_ and *R*_ct_, which is determined by the diameter of the semicircle, was measured as 9.65 Ω for Cu‐SiO*_x_*‐1@PCNF‐1200 and 4.75 Ω for SiO*_x_*‐1@PCNF‐1200, indicating lower resistance when the freestanding electrode was used (Figure S11, Supporting Information). Similarly, after lithiation, *R*_ele_ remained consistent for both electrodes, whereas *R*_tot_ was measured as 3.25 and 9.24 Ω for Cu‐SiO*_x_*‐1@PCNF‐1200 and SiO*_x_*‐1@PCNF‐1200, respectively (Figure S11, Supporting Information). These results demonstrate that even after lithiation, the freestanding electrode exhibited significantly lower resistance. These findings highlight that the use of the freestanding electrode reduces the interfacial resistance between the current collector and electrode, thereby enhancing the electrochemical performance. In practice, the asymmetric cells employing the Cu‐SiO*_x_*‐1@PCNF‐1200 electrodes exhibited inferior electrochemical performance compared to those with the freestanding SiO*_x_*‐1@PCNF‐1200 electrodes (Figure S12, Supporting Information), with lower CEs of 97% and a shorter cycle stability of 73 cycles for the former. This demonstrates that utilizing freestanding electrodes not only offers advantages in processing but also reduces the interfacial resistance, thereby enhancing the electrochemical performance of the cells.

**Figure 6:**
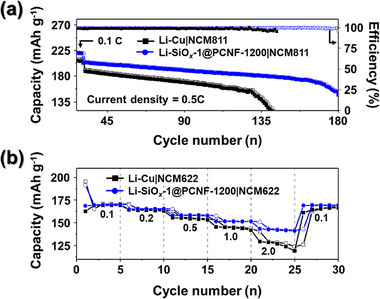
a) Cycling stability of Li‐Cu|NCM811 and Li‐SiO*_x_*‐1@PCNF‐1200|NCM811 full cells at 0.5 C and (b) rate capability at various current densities of Li‐Cu|NCM622 and Li‐SiO*_x_*‐1@PCNF‐1200|NCM622 full cells from 0.1 to 2.0 and back to 0.1 C.

Finally, to evaluate the commercial viability of the Li‐SiO*_x_*‐1@PCNF‐1200 anode, full cells were assembled using commercial cathode materials, NCM622 and NCM811, and the electrochemical performance of the cells was assessed. Similar to the symmetric cell tests, the characteristics were compared with those of Li‐Cu, and the paired full cells were named according to the same conventions, such as Li‐Cu|NCM811. The cycle stability was evaluated by pairing the anodes with the NCM811 cathodes in full cells. The N/P ratio, defined as the capacity ratio of the anode to the cathode, was set as 4.76. To ensure the formation of a stable cathode electrolyte interphase (CEI), the cells were first cycled at 0.1 C (1.0 C = 200 mA h g^−1^) for 2 cycles, followed by cycle tests at 0.5 C, as shown in **Figure** **6**a. At 0.1 C, the initial discharge capacities of Li‐Cu|NCM811 and Li‐SiO*_x_*‐1@PCNF‐1200|NCM811 were 214 and 220 mAh g^−1^, respectively. Increasing the current density to 0.5 C led to a decrease in the capacity to 188 and 204 mAh g^−1^, respectively. Across all current densities, Li‐SiO*_x_*‐1@PCNF‐1200|NCM811 consistently exhibited higher capacities compared to Li‐Cu|NCM811. Li‐Cu|NCM811 exhibited 95% capacity retention of over 142 cycles before undergoing a rapid capacity decline. In contrast, Li‐SiO*_x_*‐1@PCNF‐1200|NCM811 maintained a capacity retention of 98% over the same period, demonstrating significantly more stable cycle performance. Furthermore, the average CE of Li‐SiO*_x_*‐1@PCNF‐1200|NCM811 was 99.5%, which is notably higher that that (98.5%) of Li‐Cu|NCM811. Full‐cell tests were also conducted under harsh conditions using NCM622 cathodes, where a higher cathode loading mass was employed to achieve a lower N/P ratio (1.78). To evaluate the rate capabilities of the different full cells, the current density was incrementally increased from 0.1 C (1.0 C = 170 mA h g^−1^) to 2.0 C (Figure 6b). The Li‐Cu|NCM622 full cell exhibited reversible capacities of 169, 163, 153, 142, and 119 mAh g^−1^ at current densities of 0.1, 0.2, 0.5, 1.0, and 2.0 C, respectively. In comparison, the Li‐SiO*_x_*‐1@PCNF‐1200|NCM622 full cell showed reversible capacities of 169, 165, 158, 151, and 141 mAh g^−1^ under the same conditions. Similar to the results of the symmetric cell test presented in Figure 6b, both cells demonstrated comparable capacities at lower current densities; however, the Li‐SiO*_x_*‐1@PCNF‐1200|NCM622 cell maintained higher capacities as the current density increased. Additionally, the Li‐Cu|NCM622 full cell exhibited a tendency for capacity fading at 2.0 C. Moreover, after returning the current density to 0.1 C, the capacity of the Li‐Cu|NCM622 full cell was lower than the initial value, in contrast to that of the Li‐SiO*_x_*‐1@PCNF‐1200|NCM622 cell, which retained its capacity after the rate test. To analyze the reasons for the differences in the rate performance of the two full cells, the voltage profiles at varying current densities during the rate tests are presented in Figure S13 (Supporting Information). The voltage profiles revealed that the gap between the discharge and charge profiles represents the degree of cell polarization, which is closely related to the resistance of the cell. As shown in Figure S13a (Supporting Information), at a current density of 0.1 C, the polarization of both cells was similar. However, at current densities above 0.5 C (Figure S13b–d, Supporting Information), the Cu‐Li anode exhibited significantly higher polarization than the freestanding SiO*_x_*‐1@PCNF‐1200. This observation indicates that utilizing freestanding SiO*_x_*‐1@PCNF‐1200 as 3D host structures for LMAs enables dense and uniform Li deposition and maintains the stability, even during prolonged cycles of Li deposition and stripping. Furthermore, employing a facile electrospinning process to fabricate flexible freestanding electrodes mitigated the increase in the resistance at high current densities. This method of creating freestanding electrodes, optimizing the structure of conductive carbon in the composite, and introducing polar yet insulative SiO*_x_* facilitates stable and dense lithium deposition. Such advancements are expected to contribute significantly to the commercialization of Li metal batteries (LMBs) with high energy densities.

## 3 Conclusion

This study presents a rationally designed 3D freestanding SiO*_x_*‐1@PCNF composite as an advanced Li‐metal host, fabricated via electrospinning and subsequent carbonization. The entire host architecture was purposefully developed to eliminate the need for conventional current collectors and binders. By embedding both nanoscale HNCs derived from ZIF‐8 and microscale longitudinal tubular pores originating from PS within a 1D fibrous matrix, the structure enables maximum Li accommodation per geometric volume and facilitates uniform Li deposition throughout the internal voids. This hierarchical dual‐pore system enhances electrolyte infiltration and suppresses dendritic growth, supporting stable long‐term cycling under high‐capacity conditions. SiO*_x_*‐1@PCNF‐1200, comprising conductive N‐doped CNFs with uniformly embedded SiO*_x_* domains, exhibited mechanical durability and enhanced electrochemical performance. Moreover, the synergistic combination of conductive CNFs and insulating yet lithiophilic SiO*_x_* resulted in balanced electronic conductivity and uniform Li nucleation, avoiding Li crowding near the surface. As a result, Li plating at a capacity of 5.0 mAh cm^−2^ proceeded evenly across the electrode thickness, including within the inner pore network of the fibers. Electrochemical analysis confirmed the polar nature of SiO*_x_*, contributing to high specific capacitance despite the relatively low surface area of SiO*_x_*‐1@PCNF‐1200. EIS measurements further demonstrated low interfacial resistance when SiO*_x_*‐1@PCNF‐1200 was used on a Cu substrate. For practical application, the composite anode was paired with commercial NCM622, achieving a capacity of 141 mAh g^−1^ at 2.0 C under a low N/P ratio of 1.78, underscoring its viability for full‐cell integration. In summary, this work demonstrates a scalable strategy for constructing structurally optimized Li‐metal host materials that integrate hierarchical porosity, conductive frameworks, and lithiophilic domains. The SiO*_x_*‐1@PCNF‐1200 composite offers a promising pathway for the realization of stable, high‐performance LMBs, advancing their practical commercialization.

## 4 Experimental Section

### Sample Preparation

3D, freestanding, SiO*_x_*‐1@PCNF was prepared using a facile electrospinning process, followed by heat treatment. To prepare the electrospinning solution, 2.0 g of ZIF‐8 polyhedra was dispersed in 25 mL of *N,N*‐dimethylformamide (DMF, SAMCHUN, 99.5%) via ultrasonication for 6 h, followed by an additional 3 h of stirring. ZIF‐8 polyhedra were prepared according to the protocol outlined by Lee et al.^[^ ^42^ ^]^ Thereafter, 1.25 g of tetraethyl orthosilicate (TEOS, Junsei Chemical Co., Ltd., 95%) was introduced into the solution. Subsequently, 2.0 g of polyacrylonitrile (PAN, Sigma‐Aldrich, *M_w_* = 150,000) and 2.0 g of polystyrene (PS, Sigma‐Aldrich, *M_w_* ≈ 192,000) were gradually added to the TEOS/ZIF‐8/DMF solution and stirred overnight at room temperature to ensure a well‐dispersed suspension. The viscosity of the resulting electrospinning solution was measured to be ≈1800 cP using a viscometer. For the electrospinning process, the prepared solution was loaded into a 12 mL plastic syringe fitted with a 21‐gauge stainless steel needle. The electrospinning was conducted under controlled environmental conditions with a relative humidity of 15% and a temperature of 25 °C. The solution was ejected at a controlled flow rate of 3 mL h^−1^ onto a rotating drum collector covered with Al foil, operating at 180 rpm. The distance between the needle tip and collector was maintained at 20 cm, while an applied voltage of 24 kV facilitated fiber formation. The resulting TEOS/ZIF‐8/PAN/PS composite nanofibers were then subjected to a stabilization process at 150 °C for 3 d in a hot‐air oven. Finally, the stabilized nanofibers were carbonized at 1200 °C for 5 h under N_2_ atmosphere, using a consistent heating rate of 5 °C min^−1^, to obtain the desired SiO*_x_*‐1@PCNF‐1200. For comparison, different weights of TEOS were used to prepare samples with varying SiO*_x_* contents. Based on the reference TEOS content (1.25 g used to prepare SiO*_x_*‐1@PCNF‐1200), the samples prepared with 0.625 g (half of 1.25 g) and 1.875 g (1.5 times of 1.25 g) of TEOS were designated as SiO*_x_*‐0.5@PCNF‐1200 and SiO*_x_*‐1.5@PCNF‐1200, respectively. Additionally, to investigate the effect of the carbonization temperature, the nanofibers were treated at two different temperatures (800 and 1400 °C) for 5 h under N_2_ atmosphere, yielding the SiO*_x_*‐1@PCNF‐800 and SiO*_x_*‐1@PCNF‐1400, respectively. Detailed information, including characterization methods and electrochemical measurements, is provided in Supporting Information.

## Conflict of Interest

The authors declare no conflict of interest.

## Supporting information



Supporting Information

## Data Availability

The data that support the findings of this study are available from the corresponding author upon reasonable request.
