# Supplementary material for: 3D Lithiophilic Freestanding Hosts with SiO x ‐Embedded Hierarchical Porous N‐Doped Carbon Nanofibers for Dendrite‐Free Lithium Metal Batteries
Source: Small. 2025 May 27;21(30):2504223. doi: 10.1002/smll.202504223 (PMC12306413; doi:10.1002/smll.202504223)
Supplement: Supplementary file 1 — Supporting Information [file SMLL-21-2504223-s001.pdf]

NANO · MICRO
small

Supporting Information

for *Small*, DOI 10.1002/smll.202504223

3D Lithiophilic Freestanding Hosts with SiO_x-Embedded Hierarchical Porous N-Doped Carbon Nanofibers for Dendrite-Free Lithium Metal Batteries

Yeon Woo Nahm, Jae Seob Lee, Jae Hun Choi, Jung Sang Cho* and Yun Chan Kang**

((Supporting Information can be included here using this template))

Supporting Information

3D Lithiophilic Freestanding Hosts with SiO_x-Embedded Hierarchical Porous N-Doped Carbon Nanofibers for Dendrite-Free Lithium Metal Batteries

Yeon Woo Nahm, Jae Seob Lee, Jae Hun Choi, Jung Sang Cho*, and Yun Chan Kang**

Y. W. Nahm, J. S. Lee, J. H. Choi*, Y. C. Kang*
Department of Materials Science and Engineering
Korea University
Anam-Dong, Seongbuk-Gu, Seoul 136-713, Republic of Korea
E-mail: hun7516@naver.com (J. H. Choi), yckang@korea.ac.kr (Y. C. Kang)

J. S. Lee, J. S. Cho*
Department of Engineering Chemistry
Chungbuk National University
Chungbuk 28644, Republic of Korea
E-mail: jscho@cbnu.ac.kr (J. S. Cho)

J. S. Cho*
Biomedical Research Institute, Chungbuk National University Hospital, Chungbuk 28644, Republic of Korea
Advanced Energy Research Institute, Chungbuk National University, Cheongju, Chungbuk 28644, Republic of Korea
E-mail: jscho@cbnu.ac.kr

Keywords: lithium metal battery, electrospinning, 3D host materials, hollow N-doped nanocages, zeolitic imidazolate framework-8, freestanding

Experimental Section

Characterization: The morphologies of the composite nanofibers were examined using field-emission scanning electron microscopy (FE-SEM, ULTRA PLUS, ZEISS) and field-emission transmission electron microscopy (FE-TEM, JEOL, JEM-2100F) at the Korea Basic Science Institute (KBSI), Daegu Center. Thermogravimetry-mass spectrometry (TG-MS, STA 409 PC + QMS 403 C, NETZSCH) was conducted under an N_2 atmosphere over a temperature range of 25-1200 °C at a heating rate of 10 °C min^{-1} to trace the thermal decomposition process of the composite nanofibers. The phase composition and crystal structure of the synthesized materials were analyzed via X-ray diffraction (XRD) using an Empyrean diffractometer equipped with Cu-K_{α} radiation (λ_{avg} = 1.5425 Å), operated at 40 kV and 30 mA, at KBSI Daegu. The chemical states and electronic structure of the samples were investigated by X-ray photoelectron spectroscopy (XPS, K-Alpha, Thermo Fisher Scientific), employing microfocus monochromated Al K_{α} radiation at 12 kV and 20 mA. Raman spectroscopy (Horiba Jobin Yvon, HR800, LabRam) was used to assess the crystallinity of the carbonaceous matrix in the nanofibers. The Brunauer-Emmett-Teller (BET) method was used to determine the specific surface areas of the samples using N_2 as the adsorbate gas. The thermal stability and carbon content were evaluated via thermogravimetric analysis (TGA, Pyris 1, PerkinElmer) over the temperature range of 25–1000 °C under air atmosphere at a heating rate of 10 °C min^{-1}. Four-point probe (AiT, CMT-SR2000N) was used to measure the electrical conductivity of the electrodes at the range of 5 μS cm^{-1} to 100 kS cm^{-1}.

Electrochemical measurements: CR2032 coin-type cells were assembled in an Ar-filled glove-box prior to evaluating the electrochemical properties of the samples. Lithium bis(trifluoromethane) sulfonamide (LiTFSI; 1.0 M) was dissolved in a mixture of 1,2-dimethoxyethane (DME) and 1,3-dioxolane (DOL) (1:1 in volume) with 0.3 M $LiNO_3$ to prepare the electrolyte used to test the half and symmetrical cells. The half-cell test was initialized with two precycle steps in the potential range of 0–1.0 V at 1.0 mA cm^{-2}, to stabilize the solid electrolyte interphase (SEI) and eliminate the contaminated surface. Before the symmetrical and full cell tests, Li was deposited on the working electrode at a current density of 2.0 mA cm^{-2} and total capacity of 5.0 mAh cm^{-2}. For the full cells, a slurry consisting of $LiNi_{0.6}Co_{0.2}Mn_{0.2}O_2$ (NCM622) commercial powder, polyvinylidene fluoride (PVDF), Super-P with a weight ratio of 94:3:3, and *N*-methyl-2-pyrrolidone (NMP) was cast onto Al foil to form the cathode. In addition, $LiNi_{0.8}Co_{0.1}Mn_{0.1}O_2$ (NCM811) cathodes were prepared by mixing the active material, PVDF, and Super-P in a mass ratio of 8:1:1 in NMP. The mass

loading of the cathode was 16.5 and 5.2 mg cm^{-2} for NCM622 and NCM811 respectively. $LiPF_6$ (1 M) was added to a solution of ethylene carbonate (EC) and ethyl methyl carbonate (EMC) (3:7 in volume) with 2 wt% fluoroethylene carbonate (FEC) to prepare the electrolytes used in the full cells. NCM622 had a theoretical capacity of 170 mAh g^{-1}, calculated cathode capacity of 2.80 mAh cm^{-2}, and NCM811 had calculated capacity of 1.05 mAh cm^{-2} based on the theoretical capacity of 200 mAh g^{-1}. The capacity of Li deposited on the anode was 5.0 mAh cm^{-2}, the N/P ratio of the full cells with NCM622 and NCM811 was 1.78 and 4.76, respectively. Electrochemical impedance spectroscopy (EIS) analyses were performed in the frequency range of 0.01 Hz to 1000 kHz. Cyclic voltammetry (CV) tests were conducted within the voltage range of 0.0–1.5 V at a scan rate of 0.1–2.0 mV s^{-1}.

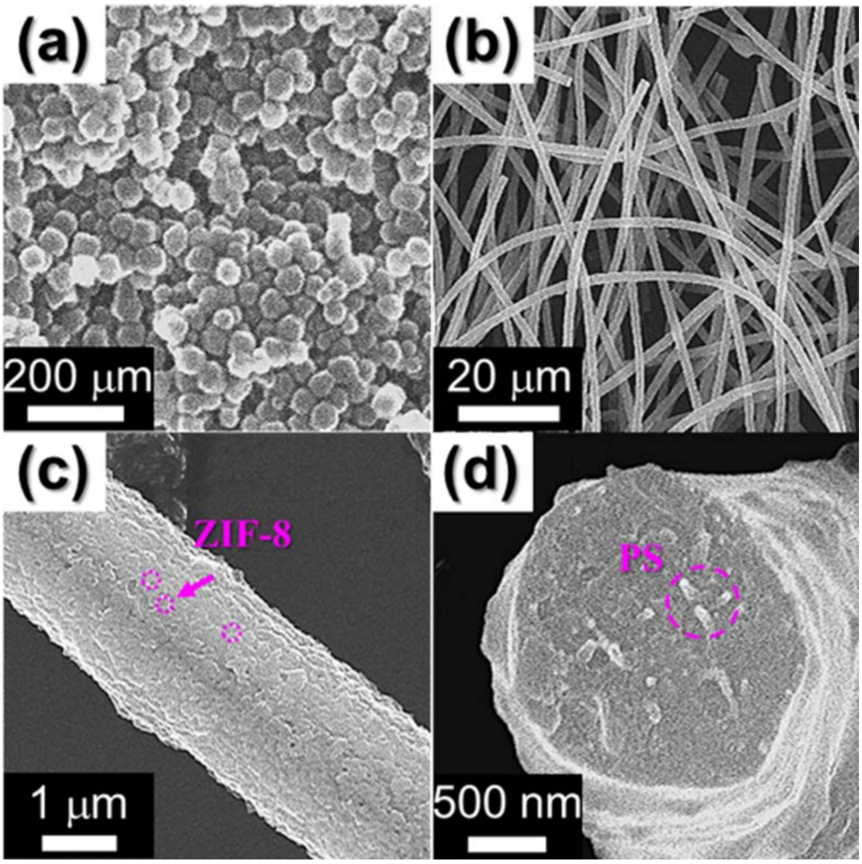

Figure S1. FE-SEM images of a) ZIF-8 polyhedra, and b-d) as-spun TEOS/ZIF-8/PAN/PS fibers.

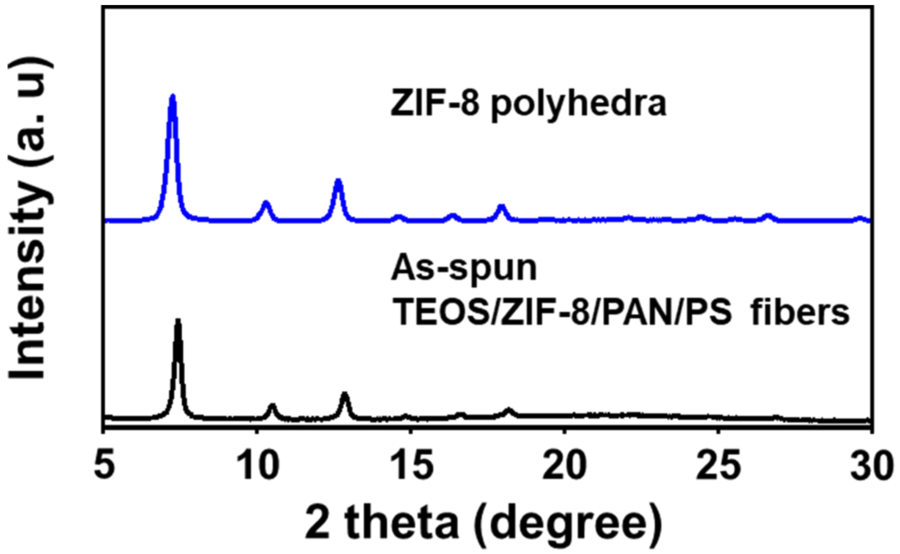

Figure S2. XRD patterns of ZIF-8 polyhedra and as-spun TEOS/ZIF-8/PAN/PS fibers.

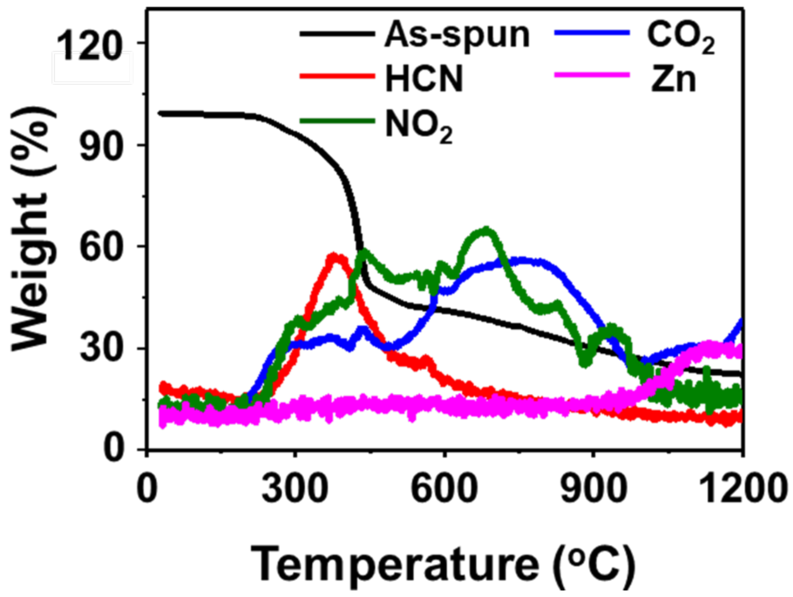

Figure S3. TG-MS curves performed under N_2 atmosphere of the as-spun TEOS/ZIF-8/PAN/PS fibers obtained after stabilization at 150 °C.

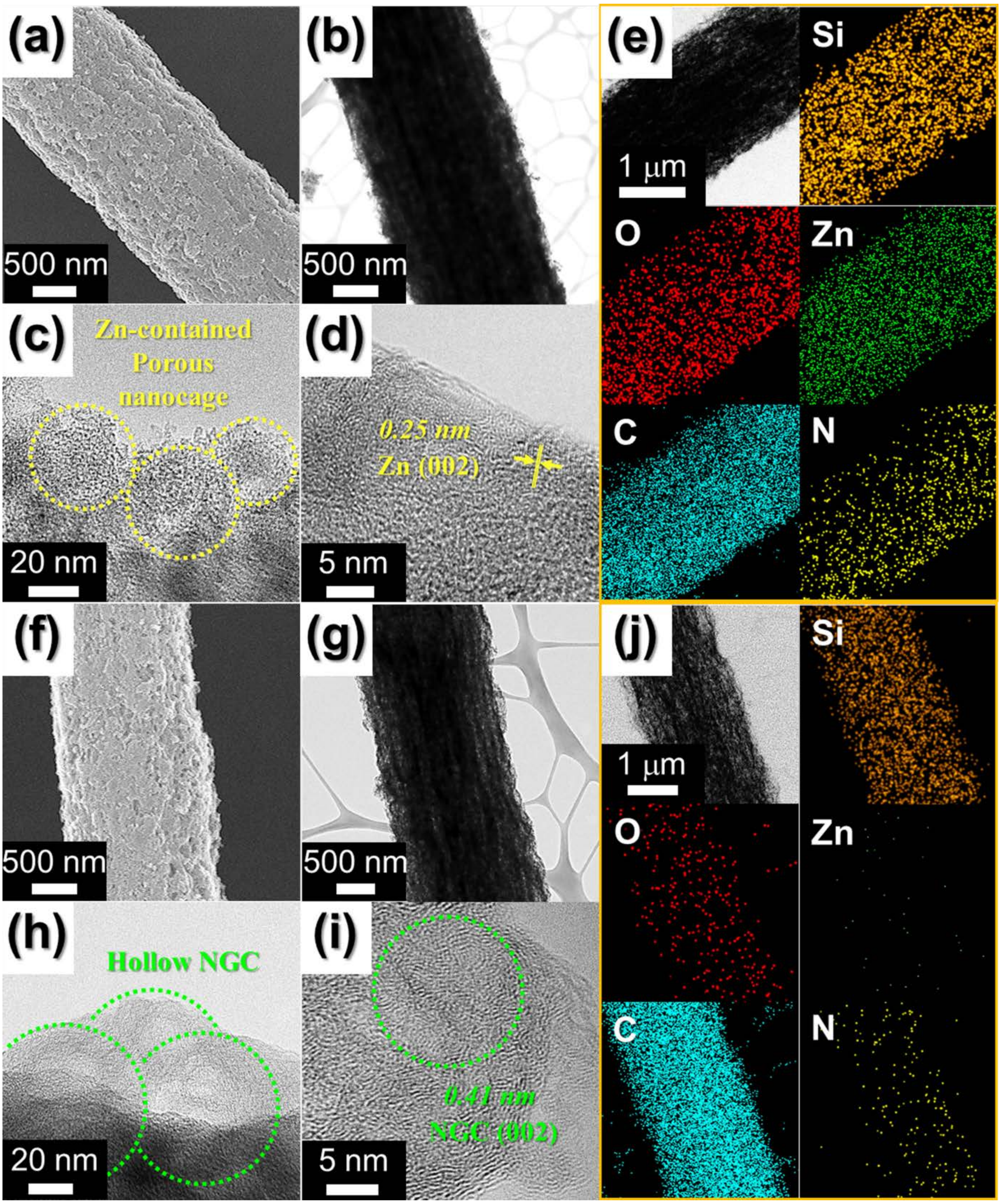

Figure S4. Morphologies and elemetal mapping images of the a-e) SiO_x-1@PCNF-800, f-j) SiO_x-1@PCNF-1400: a,f) FE-SEM images, b,c,g,h) TEM images, d,i) HR-TEM images, and e,j) elemetmal mapping images.

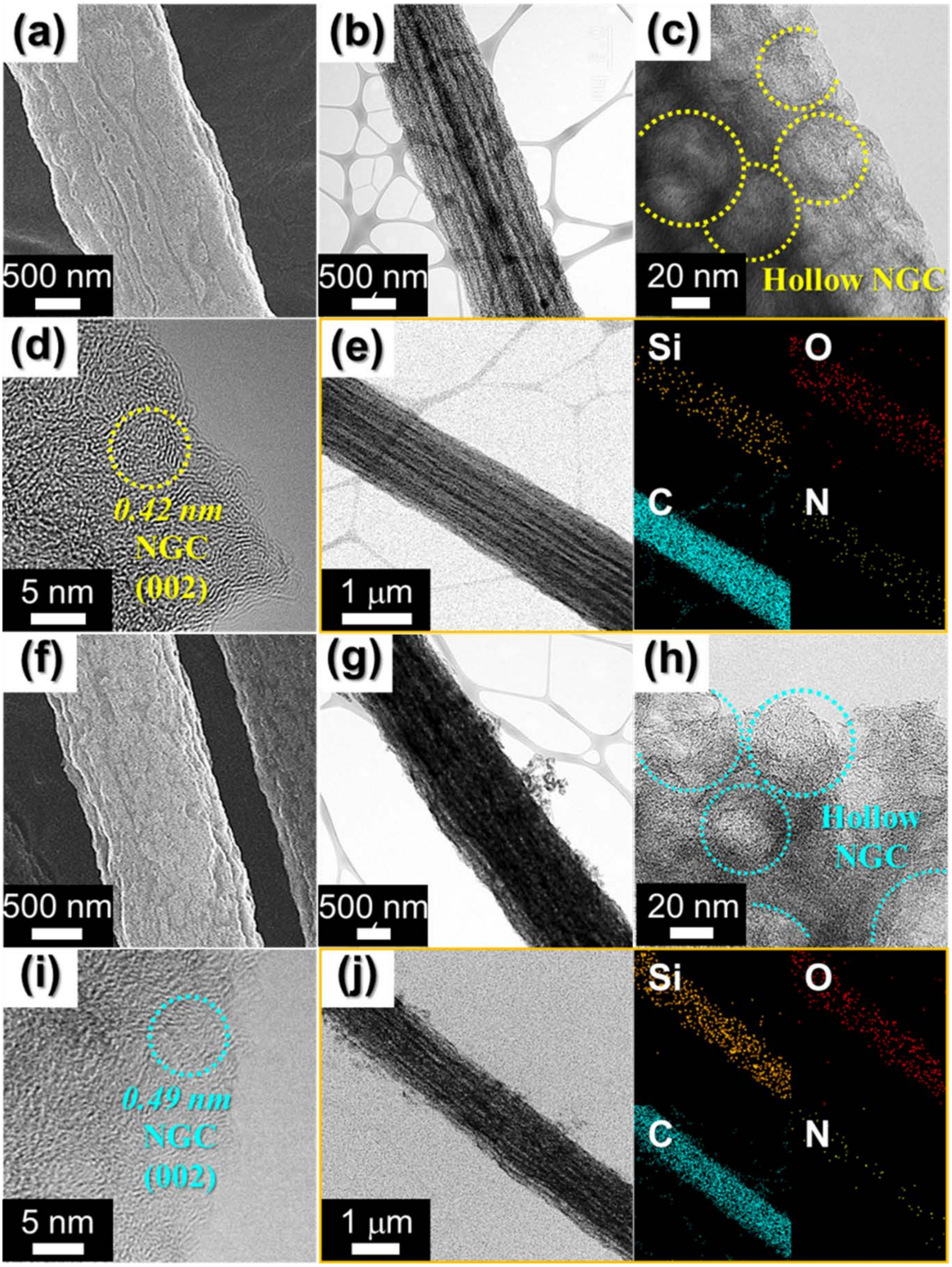

Figure S5. Morphologies and elemetal mapping images of the a-e) SiO_x-0.5@PCNF-1200, f-j) SiO_x-1.5@PCNF-1200: a,f) FE-SEM images, b,c,g,h) TEM images, d,i) HR-TEM images, and e,j) elemetmal mapping images.

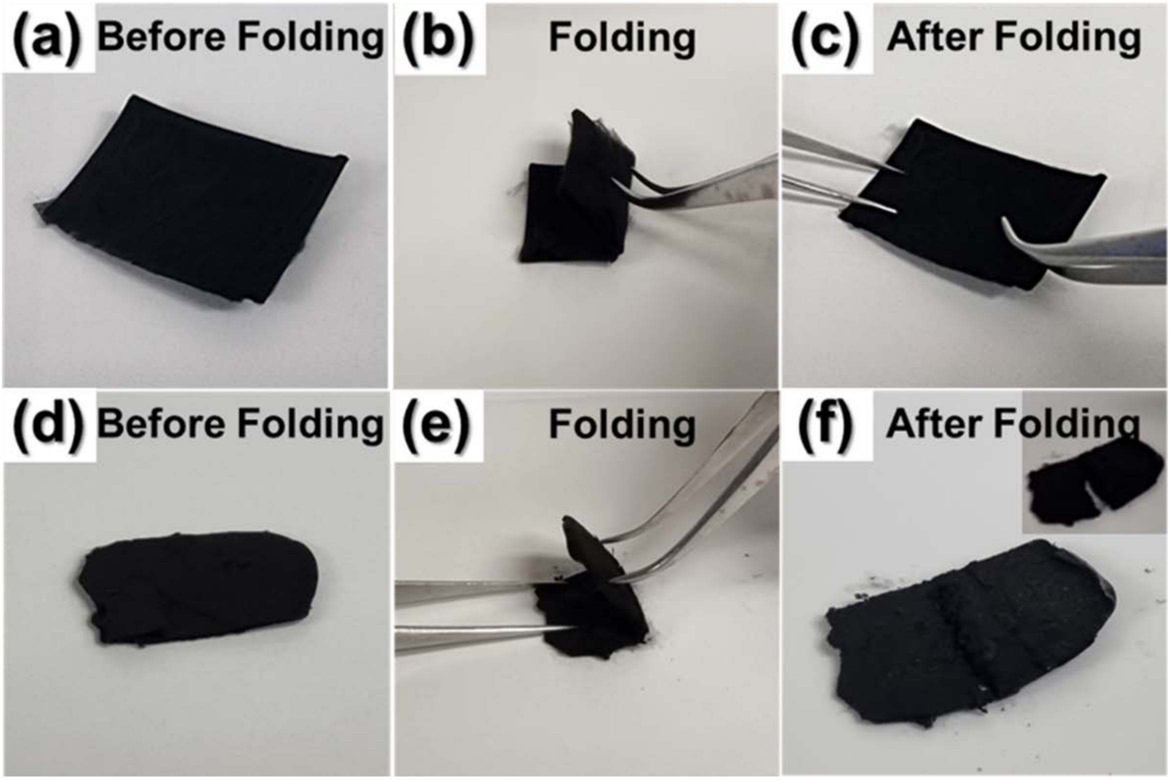

Figure S6. Flexibility test of SiO_x-1@PCNF-1200 and SiO_x-2@PCNF-1200: a,d) before folding, b,e) during folding, and c,f) after folding of freestanding sheet.

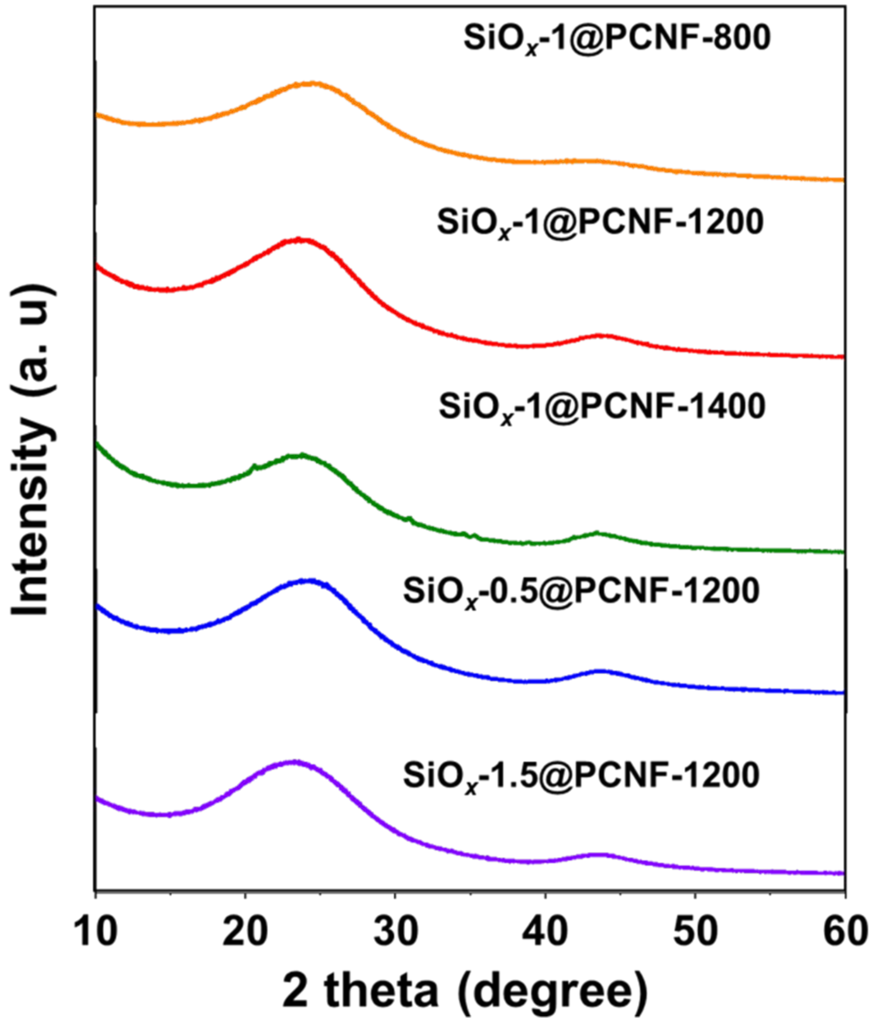

Figure S7. XRD patterns of the fibers carbonized at different temperatures (SiO_x-1@PCNF-800, -1200, -1400) and fibers with different SiO_x content SiO_x-X@PCNFs-1200 (X = 0.5, 1, 1.5).

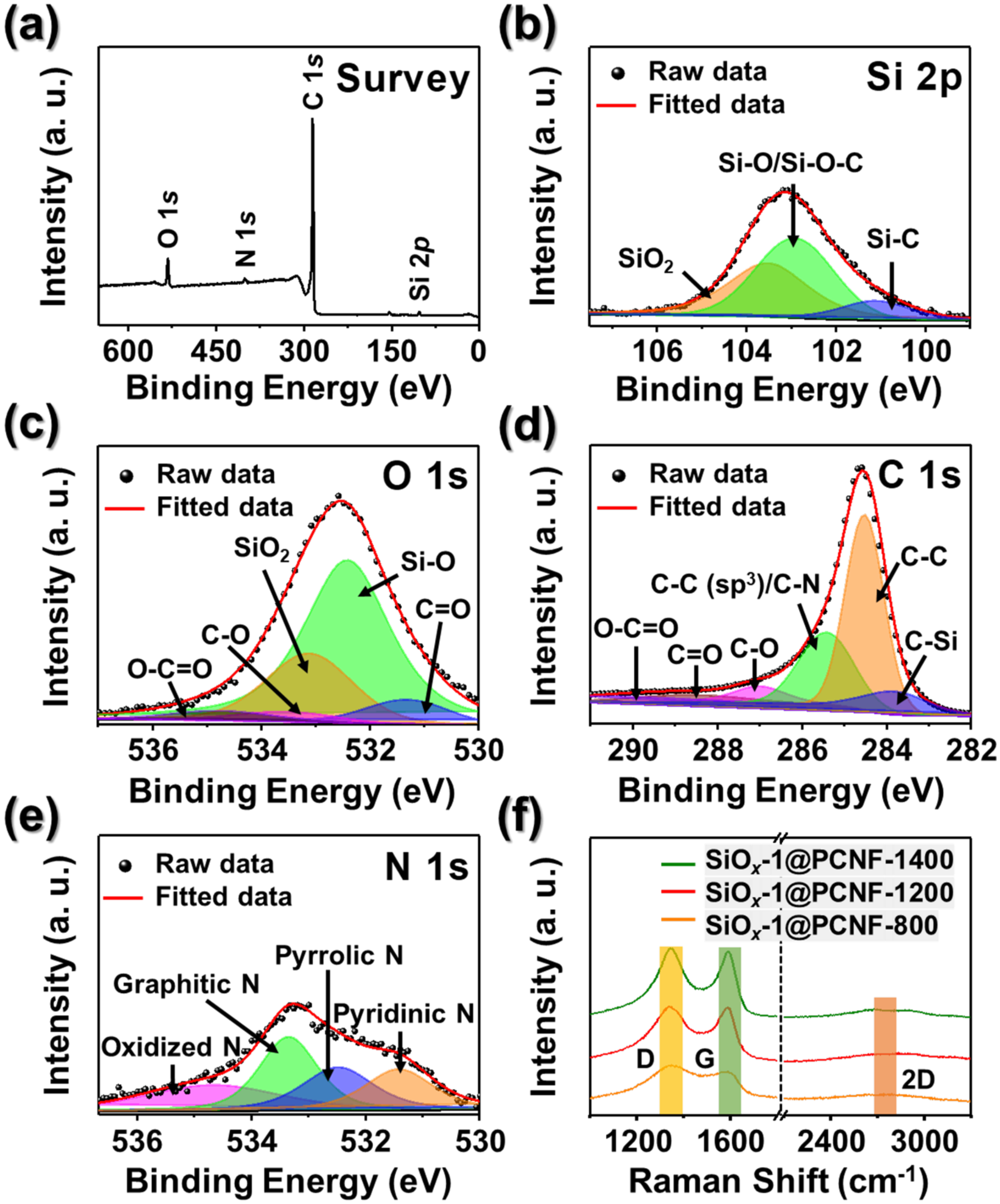

Figure S8. a) XPS spectra of SiO_x-1@PCNF-1200, b) Si 2*p*, c) O 1*s*, d) C 1*s*, e) N 1*s*, and f) Raman spectra of SiO_x-1@PCNF-800, -1200 and -1400.

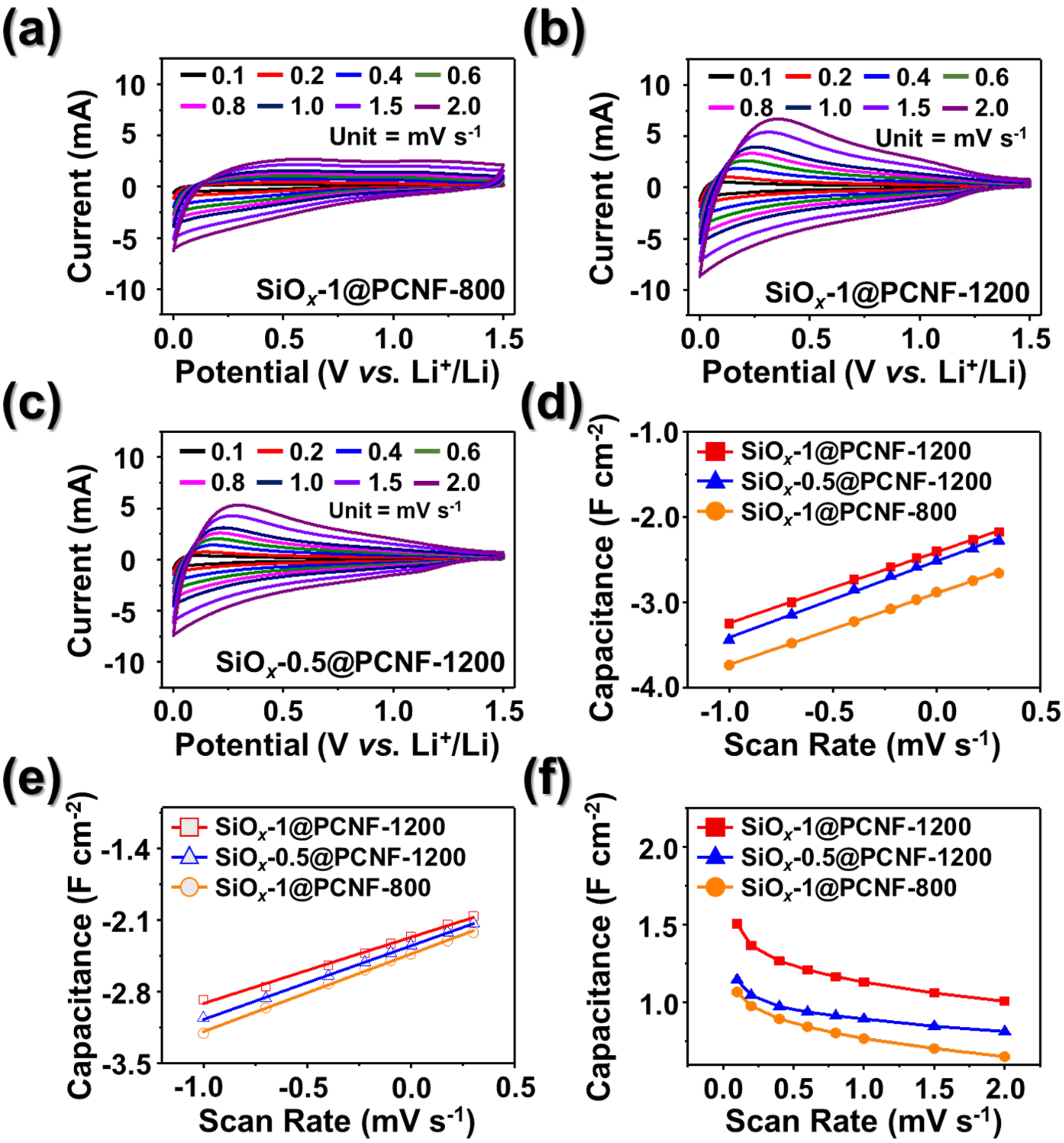

Figure S9. CVs of a) SiO_x-1@PCNF-800, b) SiO_x-1@PCNF-1200, and c) SiO_x-0.5@PCNF-1200 at various scan rates ranging from 0.1 to 2.0 mV s^{-1}. d) Log(i)–log(v) plots for the cathodic peaks used to determine the b-values for each sample. e) Log(i)–log(v) plots for the anodic peaks used to determine the b-values for each sample. f) Areal capacitances of SiO_x-1@PCNF-800, SiO_x-1@PCNF-1200, and SiO_x-0.5@PCNF-1200 as a function of scan rate.

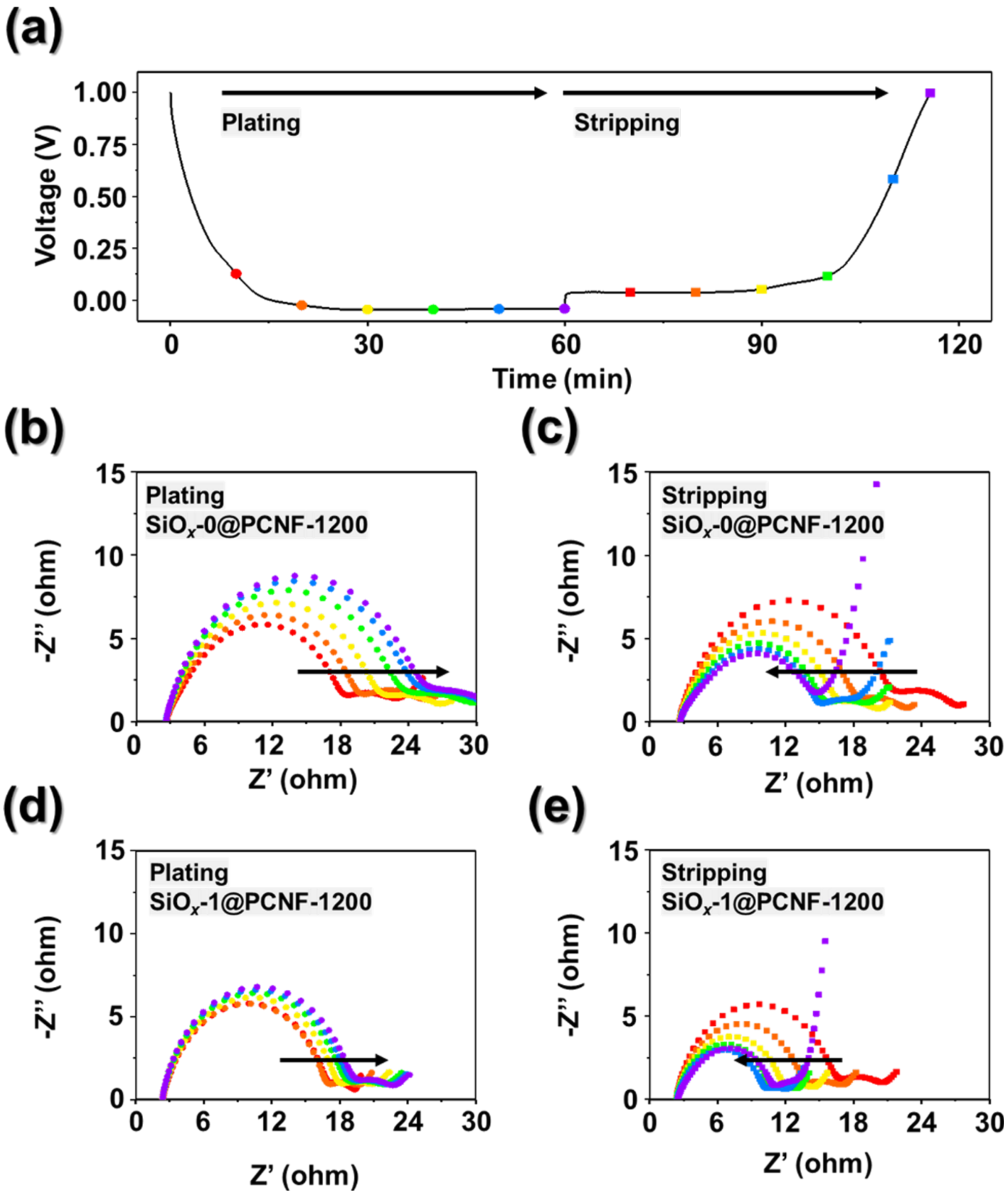

Figure S10. In-situ EIS analysis during Li plating and stripping processes in half cells. a) Voltage profile during EIS measurements, in-situ Nyquist plots of the SiO$_x$-0@PCNF-1200 electrode during the b) plating and c) stripping processes, in-situ Nyquist plots of the SiO$_x$-1@PCNF-1200 during the d) plating and e) stripping processes.

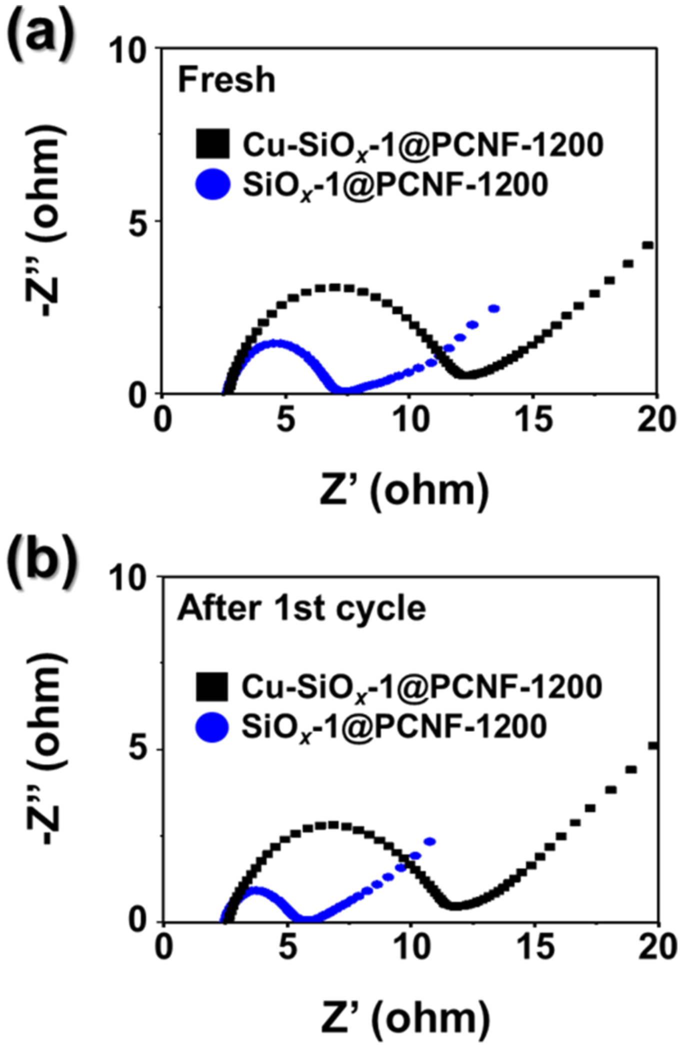

Figure S11. EIS analysis of the a) fresh cells and b) cells after 1st cycle of SiO_x-1@PCNF-1200 and Cu-SiO_x-1@PCNF-1200 at the current density of 1.0 mA cm^{-2}.

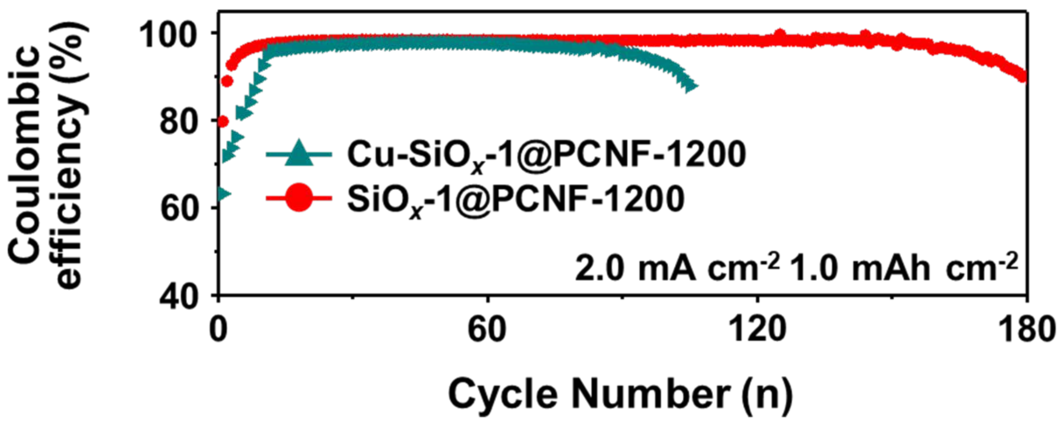

Figure S12. CEs of SiO_x-1@PCNF-1200 and Cu-SiO_x-1@PCNF-1200 at a current density of 2.0 mA cm^{-2} with a cycling capacity of 1.0 mAh cm^{-2}.

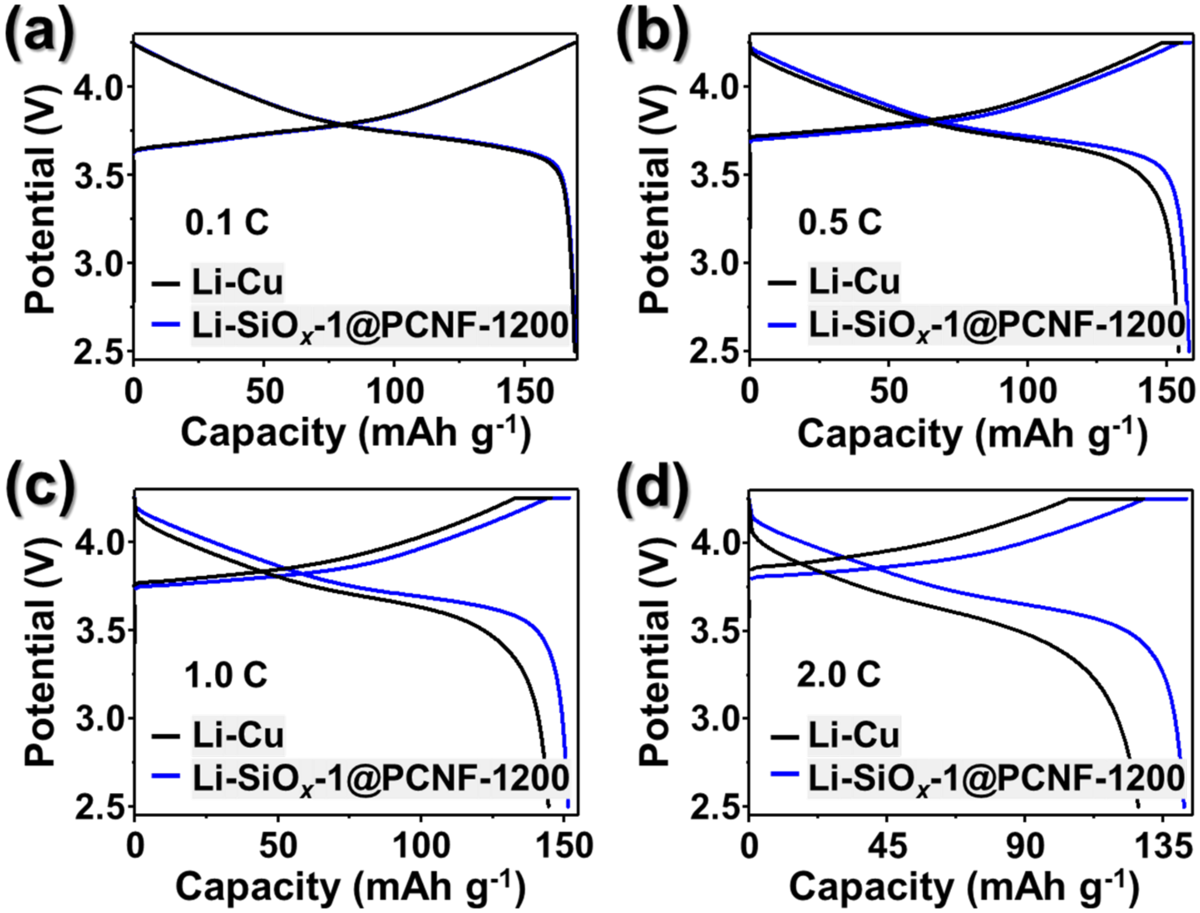

Figure S13. Voltage profiles of Li-Cu|NCM622 and Li-SiO_x-1@PCNF-1200|NCM622 full cells at the current density of a) 0.1, b) 0.5, c) 1.0, and d) 2.0 C.

Table S1. Performance comparison of asymmetric cell tests

Samples	Asymmetric Test Capacity	Asymmetric Test Current Density	Asymmetric Test Cycle	Asymmetric Test Coulombic Efficiency	Ref
**SiO_x-1@PCNF-1200**	**1.0 mAh cm^{-2}**	**2.0 mA cm^{-2}**	**160 cycles**	**98.0 %**	**This work**
MS-ZS@PHC	1.0 mAh cm^{-2}	2.0 mA cm^{-2}	160 cycles	97.0 %	[S1]
NPHCF (freestanding)	1.0 mAh cm^{-2}	1.0 mA cm^{-2}	200 cycles	94.0 %	[S2]
Sn/CNF	1.0 mAh cm^{-2}	1.0 mA cm^{-2}	150 cycles	98.0 %	[S3]
SiC/CC	1.0 mAh cm^{-2}	2.0 mA cm^{-2}	100 cycles	92.0 %	[S4]
ANCFP	1.0 mAh cm^{-2}	2.0 mA cm^{-2}	120 cycles	98.0 %	[S5]

Table S2. Electrical conductivities of the fibers carbonized at different temperatures (SiO_x-1@PCNF-800, -1200, -1400), and with different SiO_x content SiO_x-X@PCNFs-1200 (X = 0.5 and 1.5).

	SiO_x-1@PCNF-800	SiO_x-0.5@PCNF-1200	SiO_x-1@PCNF-1200	SiO_x-1@PCNF-1400	SiO_x-1.5@PCNF-1200
Conductivity (S cm^{-1})	Out of range (Too low)	1.9	0.7	2.3	0.42

Table S3. Performance comparison of symmetric cell tests.

Samples	Cycling capacity	Cycle life	Voltage Hysteresis	Ref
**SiO_x-1@PCNF-1200**	**1.0 mAh cm^{-2}**	**1350 h (1.0 mA cm^{-2})**	**28 mV (1.0 mA cm^{-2})<br>45 mV (3.0 mA cm^{-2})<br>69 mV (5.0 mA cm^{-2})<br>123 mV (10.0 mA cm^{-2})**	**This work**
SiC/CC	1.0 mAh cm^{-2}	1000 h (1.0 mA cm^{-2})	40 mV (1.0 mA cm^{-2})<br>44 mV (2.0 mA cm^{-2})<br>64 mV (4.0 mA cm^{-2})<br>102 mV (6.0 mA cm^{-2})	[S4]
ZnO@C-d-CFs	1.0 mAh cm^{-2}	1200 h (1.0 mA cm^{-2})	40 mV (1.0 mA cm^{-2})<br>80 mV (3.0 mA cm^{-2})<br>120 mV (5.0 mA cm^{-2})	[S6]
Mxene/SiO_2	1.0 mAh cm^{-2}	1000 h (1.0 mA cm^{-2})	79 mV (1.0 mA cm^{-2})	[S7]
Nb_2O_5-CNF	1.0 mAh cm^{-2}	1000 h (1.0 mA cm^{-2})	52 mV (1.0 mA cm^{-2})	[S8]
Au@PHCF	1.0 mAh cm^{-2}	1000 h (1.0 mA cm^{-2})	30 mV (1.0 mA cm^{-2})	[S9]
CBG	1.0 mAh cm^{-2}	630 h (1.0 mA cm^{-2})	39 mV (1.0 mA cm^{-2})	[S10]
Co-N-CNT-CF	1.0 mAh cm^{-2}	1000 h (1.0 mA cm^{-2})	45 mV (1.0 mA cm^{-2})<br>60 mV (2.0 mA cm^{-2})<br>70 mV (4.0 mA cm^{-2})	[S11]
Cu/Cu_3P-N-CNF	1.0 mAh cm^{-2}	1494 h (1.0 mA cm^{-2})	60 mV (1.0 mA cm^{-2})	[S12]
NOCA@CF	1.0 mAh cm^{-2}	800 h (1.0 mA cm^{-2})	36 mV (1.0 mA cm^{-2})<br>106 mV (3.0 mA cm^{-2})<br>159 mV (5.0 mA cm^{-2})<br>327 mV (10.0 mA cm^{-2})	[S13]
Ag@CMF	1.0 mAh cm^{-2}	1000 h (1.0 mA cm^{-2})	49 mV (1.0 mA cm^{-2})	[S14]

References

[S1] J. Wen, X. Song, X. Li, C. Yan, J. Zou, H. Wu, Q. Zhang, X. Zeng, *J. Colloid Interface Sci.* **2022**, 622, 347.

[S2] J. Jian, Y. Zhang, J. Sun, L. Peng, C. Lu, C. Jin, H. Wang, R. Yang, *ACS Appl. Energy Mater.* **2021**, 4, 14191.

[S3] C. Fu, H. Yang, P. Jia, C. Zhao, L. Wang, T. Liu, *J. Mater. Chem. A* **2023**, 11, 15237.

[S4] B. Sun, Q. Zhang, W. Xu, R. Zhao, H. Zhu, W. Lv, X. Li, N. Yang, *Nano Energy* **2022**, 94, 106937.

[S5] Y. K. Lee, K. Y. Cho, S. Lee, J. Choi, G. Lee, H. I. Joh, K. Eom, S. Lee, *Adv. Energy Mater.* **2023**, 13, 2203770.

[S6] S. Fan, Z. Sun, C. Liu, F. Ye, M. Liu, *ChemSusChem* **2025**, e202402472.

[S7] S. Wang, Y. Li, X. Zhou, Y. Yang, G. Chen, *J. Mater. Chem. A* **2023**, 11, 20165.

[S8] S. Yi, D. Hong, Z. Su, L. Tian, W. Zhang, M. Chen, M. Hu, B. Niu, Y. Zhang, D. Long, *ACS Appl. Mater. Interfaces* **2021**, 13, 56498.

[S9] D. W. Kang, S. S. Park, H. J. Choi, J.-H. Park, J. H. Lee, S.-M. Lee, J.-H. Choi, J. Moon, B. G. Kim, *ACS Nano* **2022**, 16, 11892.

[S10] T. Li, S. Gu, L. Chen, L. Zhang, X. Qin, Z. Huang, Y. B. He, W. Lv, F. Kang, *Small* **2022**, 18, 2203273.

[S11] T. Lyu, F. Luo, Z. Wang, F. Jiang, S. Geng, Y. Zhuang, X. Lin, J. Chen, D. Wang, L. Bu, *Chem. Eng. J.* **2023**, 466, 143357.

[S12] Y. Xiang, L. Lu, W. Li, F. Yan, H. Wang, Z. Zhao, J. Li, A. G. P. Kottapalli, Y. Pei, *Chem. Eng. J.* **2023**, 472, 145089.

[S13] Y. An, Y. Tian, Y. Li, C. Wei, Y. Tao, Y. Liu, B. Xi, S. Xiong, J. Feng, Y. Qian, *Chem. Eng. J.* **2020**, 400, 125843.

[S14] Y. Fang, S. L. Zhang, Z.-P. Wu, D. Luan, X. W. Lou, *Sci. Adv.* **2021**, 7, eabg3626.
